# Endothelial cell-derived extracellular vesicles modulate the therapeutic efficacy of mesenchymal stem cells through IDH2/TET pathway in ARDS

**DOI:** 10.1186/s12964-024-01672-0

**Published:** 2024-05-27

**Authors:** Xiao Wu, Ying Tang, Xinxing Lu, Yigao Liu, Xu Liu, Qin Sun, Lu Wang, Wei Huang, Airan Liu, Ling Liu, Jie Chao, Xiwen Zhang, Haibo Qiu

**Affiliations:** 1 Jiangsu Provincial Key Laboratory of Critical Care Medicine, Department of Critical Care Medicine, School of Medicine, Zhongda Hospital, Southeast University, Nanjing, 210009 China; 2 Department of Physiology, School of Medicine, Southeast University, Nanjing, 210009 China

**Keywords:** Acute respiratory distress syndrome, Extracellular vesicles, Endothelial cells, Mesenchymal stem cells, DNA hydroxymethylation, Isocitrate dehydrogenase

## Abstract

**Background:**

Acute respiratory distress syndrome (ARDS) is a severe and fatal disease. Although mesenchymal stem cell (MSC)-based therapy has shown remarkable efficacy in treating ARDS in animal experiments, clinical outcomes have been unsatisfactory, which may be attributed to the influence of the lung microenvironment during MSC administration. Extracellular vesicles (EVs) derived from endothelial cells (EC-EVs) are important components of the lung microenvironment and play a crucial role in ARDS. However, the effect of EC-EVs on MSC therapy is still unclear. In this study, we established lipopolysaccharide (LPS) - induced acute lung injury model to evaluate the impact of EC-EVs on the reparative effects of bone marrow-derived MSC (BM-MSC) transplantation on lung injury and to unravel the underlying mechanisms.

**Methods:**

EVs were isolated from bronchoalveolar lavage fluid of mice with LPS - induced acute lung injury and patients with ARDS using ultracentrifugation. and the changes of EC-EVs were analysed using nanoflow cytometry analysis. In vitro assays were performed to establish the impact of EC-EVs on MSC functions, including cell viability and migration, while in vivo studies were performed to validate the therapeutic effect of EC-EVs on MSCs. RNA-Seq analysis, small interfering RNA (siRNA), and a recombinant lentivirus were used to investigate the underlying mechanisms.

**Results:**

Compared with that in non-ARDS patients, the quantity of EC-EVs in the lung microenvironment was significantly greater in patients with ARDS. EVs derived from lipopolysaccharide-stimulated endothelial cells (LPS-EVs) significantly decreased the viability and migration of BM-MSCs. Furthermore, engrafting BM-MSCs pretreated with LPS-EVs promoted the release of inflammatory cytokines and increased pulmonary microvascular permeability, aggravating lung injury. Mechanistically, LPS-EVs reduced the expression level of isocitrate dehydrogenase 2 (IDH2), which catalyses the formation of α-ketoglutarate (α-KG), an intermediate product of the tricarboxylic acid (TCA) cycle, in BM-MSCs. α-KG is a cofactor for ten-eleven translocation (TET) enzymes, which catalyse DNA hydroxymethylation in BM-MSCs.

**Conclusions:**

This study revealed that EC-EVs in the lung microenvironment during ARDS can affect the therapeutic efficacy of BM-MSCs through the IDH2/TET pathway, providing potential strategies for improving the therapeutic efficacy of MSC-based therapy in the clinic.

**Supplementary Information:**

The online version contains supplementary material available at 10.1186/s12964-024-01672-0.

## Background

Acute respiratory distress syndrome (ARDS) is a life-threatening condition characterized by increased pulmonary capillary permeability, infiltration of inflammatory cells, and diffuse alveolar oedema [1–3]. It has high morbidity and mortality among critically ill patients, with a reported mortality rate of 46% in patients with severe ARDS [4]. Current management relies primarily on supportive treatments [5–7], as no effective therapies have been established. Therefore, novel approaches are urgently needed to address this devastating disease.

Mesenchymal stem cells (MSCs) are non-haematopoietic stem cells that can self-renew and undergo multipotent differentiation. MSC-based therapy has shown remarkable efficacy in experimental models of acute lung injury (ALI) due to the multiple physiological effects of MSCs, including anti-inflammatory, antiapoptotic, and immunomodulatory effects [8, 9]. However, while clinical investigations of MSC administration in ARDS have shown that MSC treatment is safe, they have yet to establish its efficacy [10, 11]. Notably, interactions between MSCs and the local lung microenvironment play a crucial role in determining the efficacy of MSCs [12, 13]. Animal studies have revealed that the administration of MSCs has a protective effect against ARDS in the presence of low concentrations of the inflammatory cytokine interleukin 6 (IL-6), coagulation factors, and the fibronectin, while it has an aggravating effect on ARDS in the presence of high concentrations of IL-6 and fibronectin, as well as low total antioxidant capacity [12]. Notably, human bone marrow-derived MSCs (hMSCs) exposed to in vitro culture conditions and bronchoalveolar lavage fluid (BALF)samples obtained from ARDS patients exhibited differential expression of genes encoding known MSC-secreted mediators, including angiopoietin 1, fibroblast growth factor 7 (FGF-7), and IL-6 [14]. Therefore, changes in the lung microenvironment have been shown to affect MSC behaviours.

Extracellular vesicles (EVs), as important components of the lung microenvironment, have recently emerged as key players in the transmission of biological signals between cells [15, 16]. EVs are membrane-encapsulated particles that are released by almost all cell types under both physiological and pathophysiological conditions and mediate intercellular communication by transferring proteins, nucleic acids, and lipids from donor to recipient cells [17–19]. These events activate signal transduction pathways and deliver vesicle contents that can influence the recipient cell phenotype. Our previous study indicated that most EVs were secreted by endothelial cells (EC-EVs) or platelets in ex vivo perfused human lungs from patients with bacterial pneumonia, and these EVs aggravated lung injury, suggesting that EC-EVs play a critical role in the progression of ALI [20]. Thus, we hypothesize that the effect of MSC treatment can be influenced by EC-EVs in the lung microenvironment in ARDS patients.

Increasing evidence suggests that epigenetic regulatory mechanisms, such as DNA methylation, histone modifications, and noncoding RNAs, play key roles in MSC dysfunction, thereby affecting the therapeutic efficacy of MSCs in multiple diseases [21]. Among these epigenetic changes, DNA methylation has emerged as a significant contributor to MSC behaviours [22–24]. The ten-eleven translocation (TET) enzymes oxidizes 5-methylcytosine (5mC) to form 5-hydroxymethylcytosine (5hmC) and further oxidizes 5hmC to form 5-formylcytosine (5fC) and 5-carboxycytosine (5caC) modifications, ultimately facilitating the removal of DNA methylation products [25–27]. However, TET enzymes exhibit greater activity toward 5mC-DNA than toward 5hmC/5fC-DNA [28]. Thus, the generation of 5hmC has been identified as an important mechanism for DNA demethylation. Previous studies have demonstrated a significant decrease in *Tet1* and *Tet2* expression in bone marrow-derived MSC (BM-MSC)-deficient mice, particularly in ovariectomized mice, accompanied by a decrease in 5hmC levels. Overexpression of *Tet2* can significantly improve osteogenic and lipogenic differentiation [29], while depletion of *Tet1* and *Tet2* in BM-MSCs results in reduced exosome release [30]. Taken together, these findings suggest a novel role of TET-mediated DNA hydroxymethylation in MSC-based therapy. A deeper understanding of the regulation of DNA hydroxymethylation in MSCs will provide valuable insights into cell transplantation-based therapeutics for ARDS. However, whether EC-EVs have regulatory effects on DNA hydroxymethylation in MSCs has not been determined.

In the present study, we observed a significant increase in the number of EC-EVs in the BALF of both ALI mice and ARDS patients, and ARDS patients exhibited a more pronounced increase in the number of EC-EVs than ALI mice. Furthermore, treatment with EVs isolated from lipopolysaccharide (LPS)-treated immortalized mouse pulmonary microvascular endothelial cells (iMPMECs) (LPS-EVs) reduced the viability and migration of BM-MSCs and thus reversed the therapeutic effects of BM-MSCs on ALI/ARDS. Mechanistically, LPS-EVs reduced the expression level of isocitrate dehydrogenase 2 (IDH2) in BM-MSCs, leading to a decrease in the formation of α-ketoglutarate (α-KG), a cofactor for the TET enzymes, and consequently decreased TET activity in BM-MSCs, resulting in a decrease in MSC reparative capability. Our results provide new insights into the metabolic-epigenetic modulation of BM-MSC function by EC-EVs in ARDS. Additionally, these findings may help to elucidate the differential therapeutic effects of MSCs between humans and mice, thus providing new directions for enhancing the efficacy of MSC-based therapies.

## Materials and methods

### Animal model of LPS-induced ALI and extraction of BALF

Male C57BL/6J wild-type mice (6–8 weeks old) were purchased from GemPharmatech Co., Ltd. (Nanjing, China). All mice were maintained in a specific pathogen–free facility in the Animal Center of Southeast University. All mice were anesthetized with isoflurane (RWD, China). LPS (5 mg/kg) (*Escherichia coli* 0111:B4, Sigma, USA) was administered intratracheally to induce direct lung injury (ALI group; *n* = 12), and negative control mice received an equal volume of PBS (Sham group; *n* = 12). Twenty-four hours after sham or ALI surgery, the mice were anaesthetized, and the BALF was extracted as previously described [31]. In brief, first, the mouse was placed on its back on an operating table. After disinfecting the coat with 70% ethanol, a vertical incision of the skin above the thymus was made, and two tweezers were used to carefully pull the tissue apart until the oesophagus and the trachea were visible. Then, a tiny horizontal incision was made between the two tracheal rings. The tracheal tube was carefully placed into the cut, and the syringe was connected to the tracheal tube. The lung was slowly filled with 800 µl of PBS. This process was repeated twice, and a maximum of 2 ml of BALF was ultimately retrieved. The retrieved BALF was centrifuged at 300 × g for 5 min at 4 °C, 2,000 × g for 20 min at 4 °C, and 13,000 × g for 30 min at 4 °C to remove dead cells, cell debris, and large extracellular vesicles; the obtained fluid is referred to as conditioned mouse BALF (mBALF) in the present study.

All animal studies were performed in accordance with the National Institutes of Health Guide for the Care and Use of Laboratory Animals (National Academies Press, 2011). All procedures were approved by the Institutional Animal Care and Use Committee of the medical school, Southeast university (Approval no.20,200,301,001).

### Patient samples

BALF samples were obtained from patients who were admitted to the Department of Critical Care Medicine, Zhongda Hospital, Southeast University. Patients who met the criteria for pneumonia [32] and met the Berlin Definition [33] within 24 h were included (ARDS group) in the study. Severely immunocompromised patients and patients who had malignant tumours or who were pregnant or lactating and were excluded from this study. Critically ill patients without ARDS or pneumonia who met the above exclusion criteria were used as controls (Non-ARDS group) to eliminate the effects of mechanical ventilation, fluid therapy, and other factors following ICU admission. These controls included patients who required mechanical ventilation after routine surgeries, such as spinal surgery or uvulopalatopharyngoplasty surgery. The patients were anaesthetized, and BALF was collected by injecting a total of 100 ml of normal saline (20 ml each) into the right middle lobe or lingua. Finally, 15 ml of BALF was collected in sterile sputum collection tubes. The collected BALF was then centrifuged at 300 × g for 5 min at 4 °C, 2,000 × g for 20 min at 4 °C, and 13,000 × g for 30 min at 4 °C to remove dead cells, cell debris and large extracellular vesicles; the obtained fluid is referred to as conditioned human BALF (hBALF) in the present study.

All human subjects in this study were approved by the Independent Ethics Committee for Clinical Research of Zhongda Hospital, Affiliated to Southeast University (Approval no. 2019ZDKYSB119) and conformed to the principles outlined in the Declaration of Helsinki. All patients signed a written informed consent form before specimen collection.

### EV isolation from BALF and immunofluorescence staining for nanoflow cytometry (nFCM) analysis

The mBALF and hBALF samples were centrifuged at 200,000 × g for 2 h at 4 °C with a Type 100Ti rotor (Beckman Coulter Optima XPN-100 Ultracentrifuge, USA). The pellet was washed with 6 ml of PBS, followed by a second round of ultracentrifugation at 200,000 × g for 2 h at 4 °C. Afterwards, the supernatant was removed, and the EVs were lysed with a protein extraction kit (KeyGen Biotech, China) or resuspended in 100 µl of PBS for subsequent experiments. For immunofluorescence staining of CD31-positive EVs, EVs were isolated from mBALF (1 ml) and hBALF (1 ml) by centrifugation at 200,000 × g for 2 h at 4 °C (Beckman Coulter, USA), and the pellet was resuspended in 100 µl of PBS. Then, 2 µl of a FITC-conjugated rat anti-mouse CD31 antibody (Biolegend, 102,405) and 5 µl of a FITC-conjugated mouse anti-human CD31 antibody (Biolegend, 303,103) were added, respectively. The mixtures were incubated at 37 °C for 30 min, after which the unbound antibodies were removed by washing with 6 ml of PBS and ultracentrifugation at 200,000 × g for 2 h at 4 °C. The resulting pellet was resuspended in 100 µl of PBS for nFCM analysis. Light exposure was avoided during immunofluorescence staining.

### Cell culture

iMPMECs were generated in our laboratory [34]. The cells were used at passages 3–10, and maintained in DMEM-F12 (Gibco, USA) supplemented with 5% foetal bovine serum (FBS) (ExCell, China), 1% penicillin and streptomycin (Gibco, USA), 1% endothelial cell growth supplement (ECGS) (ScienCell, USA), 100 IU/ml heparin (Solarbio, China), and 92 mg/L D-valine (Sigma, USA) and incubated at 37 °C in 5% CO_2_. The cells were grown to 70–80% confluence, washed 3 times with PBS, and treated with or without 1 µg/ml LPS (Sigma, USA) for 24 h in conditioned culture medium (CCM) containing exosome-depleted FBS. CCM was collected after 24 h. Exosome-depleted FBS was prepared by centrifuging FBS for at least 18 h overnight at 100,000 × g at 4 °C, after which the centrifuged FBS were passed through a 0.22 μm filter (Millipore, USA). MSCs derived from the bone marrow of C57BL/6 mice (Cyagen Biosciences, China) were used at passages 3–8 and cultured in DMEM-F12 supplemented with 10% FBS and 1% penicillin and streptomycin, and these cells were tested for their osteogenic, adipogenic, and chondrogenic differentiation potentials to ensure they met the characteristic of stem cells (Supplementary Fig. S1).

### iMPMEC-EV isolation

CCM was harvested from iMPMECs and centrifuged at 300 × g for 5 min at 4 °C and 2,000 × g for 20 min at 4 °C to remove dead cells and cell debris. The supernatant (100 ml) was collected and centrifuged at 13,000 × g at 4 °C for 30 min to remove large extracellular vesicles. The CCM was then ultracentrifuged at 200,000 × g at 4 °C for 2 h with a Type 70Ti rotor (Beckman Coulter Optima XPN-100 Ultracentrifuge, USA) to pellet the extracellular vesicles. The supernatant was carefully removed, and the precipitate was resuspended in 1 ml of ice-cold PBS and pooled. A second round of ultracentrifugation (200,000 × g at 4 °C for 2 h with a Type 100Ti rotor) was carried out, and the resulting precipitate was resuspended in 100 µl of sterile PBS for cell experiments. EVs produced by normal iMPMECs were referred to as “Control-EVs”, while EVs produced by iMPMECs stimulated with LPS were referred to as “LPS-EVs”.

### EV characterization

1. Nanoparticle tracking analysis (NTA): The particle size and number of purified EVs were analysed via nanoparticle tracking using a nanoparticle tracking analyser (Particle Metrix ZetaView®, Meerbusch, Germany). EVs were diluted in PBS (1:1000) and gently vortexed before being introduced into the sample chamber using a syringe pump. The nanoparticle tracking analyser used a 488 nm excitation laser and were precalibrated for size and concentration with a 100 nm PS bead reference standard. All NTA measurements were carried out using exactly the same camera settings and tracking parameters, employing values recommended for EV detection.
2. Transmission electron microscopy (TEM): For the TEM assay, EVs were purified and resuspended in PBS. Then, one drop of resuspended EVs was dropped onto a copper mesh and incubated at room temperature for 5 min. The excess liquid was then removed with filter paper. The copper mesh was stained with 1% phosphotungstic acid and incubated at room temperature for 1 min. The staining solution was blotted on one side with filter paper, and the samples were dried for 20 min at room temperature. The EVs were imaged using a transmission electron microscope (FEI, Tecnai G2 Spirit BioTwin, USA).
3. Western blot (WB) analysis: The expression of EV markers, such as the transmembrane protein CD63 (Abcam, USA), the cytosolic protein TSG101 (Santa Cruz, USA), and ALIX (Abcam, USA), and the negative control Calnexin (Cell Signaling Technology, USA), were analysed via Western blotting. The protein concentration of the EVs was quantified with a BCA Protein Assay Kit (Beyotime, China) in accordance with the manufacturer’s protocol.

### Uptake of labelled iMPMEC-EVs by BM-MSCs

For in vitro EV uptake studies, EVs were tagged with DiD (Invitrogen, USA) following the manufacturer’s instructions. Briefly, EVs were suspended in PBS supplemented with 0.1% BSA and incubated with DiD for 15 min at room temperature. A control was prepared similarly using PBS containing 0.1% BSA without EVs. The unbound dye was washed twice by ultracentrifugation at 200,000 × g for 90 min at 4 °C. The final pellet was reconstituted in PBS for subsequent analysis.

BM-MSCs were plated on glass bottom cell culture dishes (Nest, China) (2 × 10^4^ cells/dish) in complete growth medium. After 24 h, the cells were washed with serum-free culture medium, followed by treatment with DiD-labelled iMPMEC-EVs (approximately 1 × 10^3^/cell). After 16 h of treatment with DiD-labelled iMPMEC-EVs, the cells were washed with PBS, fixed with 4% paraformaldehyde, and washed three times with PBS. Then, the sections were mounted with 4,6-diamidino-2-phenylindole (DAPI) Fluoromount-G® (SouthernBiotech, USA) and imaged using a Leica Thunder Imager (Germany).

For the dynamic uptake assay, BM-MSCs were plated in a 12-well plate (Corning, USA) at 2 × 10^4^ cells/well and cultured for 24 h. Dynamic images and videos were captured with a Biotek Cytation 5 imaging reader (USA) immediately after coculture with iMPMEC-EVs. DiD labelling was performed according to the manufacturer’s instructions. To remove the unbound dye, DiD-labelled iMPMEC-EVs were ultracentrifuged at 200,000 × g at 4°C for 2 h, followed by passage through a 0.22 μm filter (Millipore, USA).

### Cell viability assay

The viability of the BM-MSCs was detected with Cell Counting Kit-8 (CCK-8) (Glpbio, China) according to the manufacturer’s protocol. In brief, cells were plated in 96-well plates (Corning, USA) (5 × 10^3^ cells/well) and allowed to grow for 24 h before treatment. The cells were then treated with different iMPMEC-EVs (approximately 1 × 10^3^/cell) or other reagents. After incubation for 24 h, cell viability was evaluated using a microplate reader (Biotek Synergy Neo2, USA) at 450 nm.

### Migration assay

The migration of BM-MSCs was assessed by scratching a confluent layer of BM-MSCs in a six-well plate (Corning, USA) using a 0–20 µl sterile pipette tip. The loose cells were removed via a PBS wash, and then, 1 ml of serum-free medium supplemented with different iMPMEC-EVs (approximately 1 × 10^3^/cell) or other reagents was added, followed by incubation at 37 °C. Images were recorded at t = 0 h and t = 12 h, after which the extent to which the wound area was reduced was determined using ImageJ analysis software (National Institutes of Health, version 1.51k) [35].

### Western blot analysis

Cell, tissue and EV lysates were prepared using lysis buffer (KeyGen Biotech, China) supplemented with PMSF (100 mM), protease inhibitors, and phosphatase inhibitors at 4 °C for 30 min and then centrifuged for 30 min at 12,000 × g at 4 °C; the supernatant containing the total protein was transferred to a new centrifuge tube. The proteins were quantified with a BCA Protein Assay Kit (Beyotime, China) in accordance with the manufacturer’s protocol. The protein samples were boiled for 10 min at 95 °C. Equal amounts of proteins were separated via SDS‒polyacrylamide gel electrophoresis (ACE Biotechnology, China), transferred to polyvinylidene difluoride (PVDF) membranes (Millipore, USA), blocked in 5% nonfat powdered milk (Beyotime, China) in TBS buffer (Solarbio, China) containing 0.1% Tween-20 (Beyotime, China) (TBS‒T), and immunoblotted with primary antibodies at 4 °C overnight (see Supplementary Table 1 for detailed information on the antibodies used). Next, the membranes were washed three times with TBS-T at room temperature for 5 min each time, followed by incubation with horseradish peroxidase (HRP)-conjugated goat anti-mouse IgG (1:5000 dilution; Proteintech, China) or HRP-conjugated goat anti-rabbit IgG (1:5000 dilution; Proteintech, China) at room temperature for 1 h. The bands were visualized using enhanced chemiluminescence reagents (Beyotime, China) on a Tanon 5200 image analyser (Tanon, China). Finally, the intensity of the bands was analysed with ImageJ software (National Institutes of Health, version 1.51k).

### qRT‒PCR analysis

Total RNA was extracted from cells using TRIzol Reagent (Invitrogen, USA). cDNA was synthesized from total RNA (1 µg) using HiScript® II Q RT SuperMix (Vazyme, China) according to the manufacturer’s instructions. Subsequently, a quantitative real-time fluorescence-based polymerase chain reaction (qRT–PCR) assay was performed using AceQ qPCR SYBR Green Master Mix (High ROX Premixed) (Vazyme, China) according to the manufacturer’s protocol. Each sample was analysed in triplicate, and the relative changes in gene expression were normalized to the expression of *Gapdh* or *Actb* and calculated by the 2(−ΔΔCt) method (see Supplementary Table 2 for details on the primer sequences used).

### Animal studies

Male C57BL/6J wild-type mice (6–8 weeks old) were anaesthetized and administered LPS (5 mg/kg) (*Escherichia coli* 0111:B4, Sigma, USA) in PBS intratracheally to induce ALI. One hour before sham or ALI surgery, the mice were intraperitoneally (i.p.) injected with one dose of GW4869 (Sigma, USA) (2.5 µg/g) to inhibit the synthesis and release of EVs [36, 37]. GW4869 was dissolved in 0.005% DMSO (Solarbio, China). Mice were randomly assigned to five groups: Sham (*n* = 6, administered two doses of 30 µl of PBS intratracheally, 4 h apart); LPS (*n* = 6, administered 5 mg/kg LPS intratracheally to establish the ALI model, then 30 µl of PBS intratracheally 4 h later); LPS + MSC (*n* = 6, 4 h after LPS administration, 5 × 10^5^ BM-MSCs were administered intratracheally in 30 µl of PBS); and LPS + MSC-Con-EVs and LPS + MSC-LPS-EVs (*n* = 6, BM-MSCs were pretreated with Control-EVs or LPS-EVs in vitro for 24 h; four hours after LPS administration, 5 × 10^5^ pretreated BM-MSCs were administered intratracheally in 30 µl of PBS). Twenty-four and 72 h after BM-MSC administration, the therapeutic effect of BM-MSCs on ALI mice was analysed.

To evaluate the reparative effects of OE-Idh2-MSCs, GW4869 was not used. Mice were randomly assigned to five groups: Sham (*n* = 6, administered two doses of 30 µl of PBS intratracheally, 4 h apart); LPS (*n* = 6, administered 5 mg/kg LPS intratracheally to establish the ALI model, then 30 µl of PBS intratracheally 4 h later); LPS + MSCs (*n* = 6, 4 h after LPS administration, 5 × 10^5^ BM-MSCs were administered intratracheally in 30 µl of PBS); LPS + Vector-MSCs (*n* = 6, 4 h after LPS administration, 5 × 10^5^ Vector-MSCs were administered intratracheally in 30 µl of PBS); and LPS + OE-Idh2-MSCs (*n* = 6, 4 h after LPS administration, 5 × 10^5^ OE-Idh2-MSCs were administered intratracheally in 30 µl of PBS). Twenty-four hours after BM-MSC administration, the therapeutic effect of the BM-MSCs on ALI mice was analysed.

### Lung histological analysis

The left lungs of euthanized mice (*n* = 3 per group at each time point) were harvested, fixed in 4% paraformaldehyde, and then embedded in paraffin. The specimens were then sectioned at a thickness of 5 μm and stained with haematoxylin and eosin. Stained sections were imaged using an Olympus SliceView VS200 stystem (Japan). The severity of lung injury was evaluated based on interstitial oedema, cellular infiltration, and parenchymal, peribronchial and perivascular haemorrhage, as described previously [38, 39]. Each criterion was graded according to a 3-point scale, with “0” representing no injury, “1” representing mild injury, “2” representing moderate injury, and “3” representing severe injury. The total lung injury score was calculated as the sum of the 3 criteria.

### Evaluation of lung oedema

The ratio of lung wet weight to body weight (LWW/BW) was measured to evaluate lung oedema [40]. Briefly, the whole lung was removed and cleared of all extrapulmonary tissues, and the LWW/BW ratio was calculated based on the lung wet weight and body weight (mg/g).

### Cytokine analysis

Mouse serum was collected on Days 1 and 3, and cytokine concentrations were compared among the groups. The concentrations of IL-1β, IL-6 and tumor necrosis factor α (TNF-α) in the serum were evaluated using ELISA kits (Elabsicence, China) according to the manufacturer’s instructions. In addition, the right lobes were processed for lung homogenization. Total RNA was subsequently extracted from the lung homogenate using TRIzol Reagent (Invitrogen, USA), and RT‒qPCR was performed to analyse the expression of *Il1b*, *Il6*, and *Tnf* as described above.

### Immunocytochemistry

The cells were fixed with 4% paraformaldehyde for 15 min and then washed with cold PBS. The cells were permeabilized with cold PBS containing 0.4% Triton X-100 (Sigma, USA) for 15 min. The permeabilized cells were then washed and blocked with 5% bovine serum albumin (BSA) (Biofroxx, Germany) in PBS containing 0.1% Triton X-100 in PBS for 1 h at room temperature before they were incubated with primary antibodies overnight at 4 °C. For 5hmC staining, permeabilized cells were treated with 2 N HCl (Merck, USA) for 30 min at 37 °C to denature the DNA and then neutralized with 100 mM Tris-HCl (pH 8.5) (Leagene Biotech, China) for 10 min before blocking. The primary antibodies used are listed in Supplementary Table 1. After being washed three times, the cells were incubated with Alexa Fluor 555-conjugated goat anti-rabbit IgG (H + L) (1:1000 dilution; Invitrogen, USA) as a secondary antibody for 2 h at room temperature in the absence of light. The nuclei were stained with DAPI Fluoromount-G®. The cells were viewed using a Leica Thunder imaging system (Leica, Germany).

### Small interfering RNA (siRNA) transfection

Small interfering RNA (siRNA) targeting *Idh2* (siIdh2) and a scramble small interfering RNA (siCtrl) were synthesized by GenePharma (see Supplementary Table 3 for details on the siRNA sequences). Cells were seeded at 2 × 10^5^ per well into 6-well Plates 1 day before transfection. Then, the cells were transfected with siIdh2 or siCtrl (all at 120 pmol/60 nM) with GP-transfect-Mate (GenePharma, China) transfection reagent. The GP-transfect-Mate/siRNA complexes were prepared in serum-free OptiMEM (Invitrogen, USA) at the recommended ratio of 1 µl of GP-transfect-Mate per 20 pmol of siRNA (1 µl). After the addition of the GP-transfect-Mate/siRNA complexes to the cells and culture for 6 h, the cell growth medium was removed, and the cells were incubated in fresh medium containing 10% FBS. Cell viability assays and migration assays were performed 48 h after transfection.

### Transduction of the lentiviral vector

BM-MSCs (5 × 10^4^ cells/well) were seeded in six-well cell culture plates and cultured at 37 °C in humidified air containing 5% CO_2_ for 24 h. The cells were grown to 20–30% confluency and transfected with empty virus (Vector group) and lentivirus overexpressing *Idh2* (OE-Idh2 group) (Genechem, China) for 16 h at a multiplicity of infection (MOI) of 50:1. Then, the stable cell lines were harvested after selection using geneticin (Genechem, China) at the minimal lethal concentration (200 µg/ml) and cultured in normal culture medium after transduction. Finally, the transduction efficiency of the BM-MSCs was evaluated by RT‒qPCR and WB analysis, as described above.

### Dot blots

Genomic DNA was extracted from BM-MSCs using a TIANamp Genomic DNA Kit (Tiangen, China) according to the manufacturer’s instructions. A NanoDrop One system (Thermo Fisher Scientific, USA) was used to quantify the DNA concentration. DNA extracts were stored at − 80 °C until use. DNA samples were loaded on nitrocellulose membranes (Beyotime, China). After being incubated at 60 °C for 1 h and blocked with 5% nonfat milk for 30 min at room temperature, the membrane was incubated with an anti-5hmC antibody (0.2 µg/ml, Abcam) at 4 °C overnight. The blots were visualized using enhanced chemiluminescence reagents (Beyotime, China) on a Tanon 5200 image analyser (Tanon, China). To ensure equal loading, the membrane was stained with methylene blue (Aladdin, China) after immunoblotting. The density of the dots was analysed with ImageJ software (National Institutes of Health, version 1.51k).

### Global 5hmC content in DNA

One or two hundred nanograms of genomic DNA was used to measure the levels of 5hmC in the DNA using the MethylFlash™ Hydroxymethylated DNA Quantification Kit (Fluorometric) (Epigentek, Farmingdale, NY) according to the manufacturer’s instructions.

### TET activity assay

A total of 2.5 micrograms of total nuclear protein isolated using EpiQuik™ Nuclear Extraction Kit I (Epigentek, Farmingdale, NY) was subjected to an Epigenase™ 5mC Hydroxylase TET Activity/Inhibition Assay Kit (Fluorometric) (Epigentek, Farmingdale, NY) according to the manufacturer’s instructions.

### α-KG assay

BM-MSCs were lysed in ice-cold α-KG assay buffer. The α-KG level was measured using an α-KG Assay Kit (Sigma Aldrich, St. Louis, MO) according to the manufacturer’s instructions.

### STRING analysis

Fifty proteins that interact with TET1, TET2, or TET3 were analysed using the String Protein Interaction Network (https://string-db.org).

### RNA-seq analysis

Total RNA was extracted from BM-MSCs pretreated with Control-EVs or LPS-EVs using TRIzol Reagent (Invitrogen, USA) according to the manufacturer’s instructions. The sample was subsequently purified using a Clean XP Kit (Beckman Coulter, USA) and an RNase-Free DNase Set (QIAGEN, Germany). An mRNA library was established using a VAHTS Universal V6 RNA-seq Library Prep Kit for Illumina® (Vazyme, China), and an Agilent 4200 bioanalyzer (Agilent Technologies, Santa Clara, CA, USA) was used to evaluate the concentration and size distribution of the cDNA library before sequencing with an Illumina NovaSeq 6000. The protocol for high-throughput sequencing was performed according to the manufacturer’s instructions (Illumina). Significantly differentially expressed genes (DEGs) were identified as those with a false discovery rate (FDR) above the threshold (Q < 0.05) and a fold change ≥ 2 using DESeq2 software [41]. A hierarchical clustering heatmap were generated for the DEGs. Gene functional enrichment analysis was performed using the Enrich R web tool.

### Statistical analysis

All the statistical analyses were performed using GraphPad Prism (v 9.0) software. Data were compared between two groups using a 2-tailed Student’s t test, and data were compared among multiple groups using one-way ANOVA followed by Tukey’s multiple comparisons test. All the experiments were performed as a minimum of 3 independent repeats. Unless stated otherwise, the data are presented as the mean ± SD. *P* < 0.05 was considered significant.

## Results

### LPS-EVs reduce the viability and migration of BM-MSCs

**Fig. 1:**
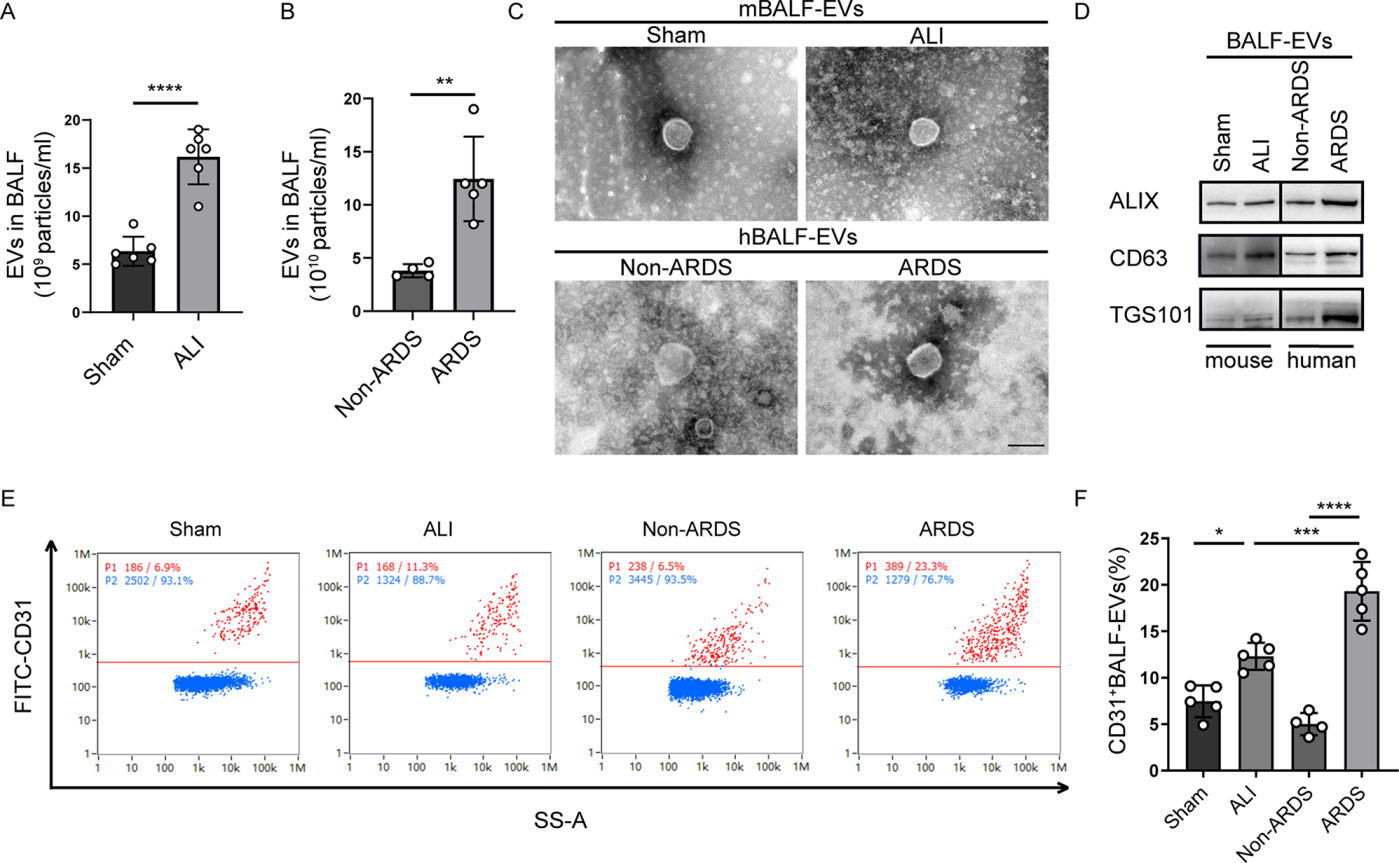
Changes of EC-EVs in the lung microenvironment of ALI mice and ARDS patients. **A, B** Nanoparticle tracking analysis (NTA) showed the number of EVs released from mouse bronchoalveolar lavage fluid (mBALF-EVs) and human bronchoalveolar lavage fluid (hBALF-EVs). **C** Representative micrographs of mBALF-EVs and hBALF-EVs observed by transmission electron microscopy (TEM). Scale bar: 100 nm. **D** Representative Western blot images for EV markers ALIX, CD63, and TSG101 in mBALF-EVs and hBALF-EVs. **E** Bivariate dot-plots of FITC fluorescence versus side-scatter(SS-A) for mBALF-EVs and hBALF-EVs upon immunofluorescent labeling with FITC-conjugated mouse antibody against CD31 and FITC-conjugated human antibody against CD31. **F** The population ratios of CD31^+^ EVs of mBALF-EVs and hBALF-EVs. (*n* = 5 for mouse sample in each group; *n* = 4 for Non-ARDS group; *n* = 5 for ARDS group.) Data are presented as mean ± SD using unpaired *t* test. *P<0.05, **P<0.01, ***P<0.001, ****P<0.0001

To evaluate the impact of EC-EVs in the lung microenvironment on the reparative effects of BM-MSC transplantation, we first aimed to determine the changes of EC-EVs in lung microenvironment during ALI/ARDS. To clarify these changes, we initially isolated EVs from mouse bronchoalveolar lavage fluid (mBALF-EVs) and human bronchoalveolar lavage fluid (hBALF-EVs) using ultracentrifugation. The demographic and clinical characteristics of the ARDS patients and non-ARDS controls are presented in Supplementary Table 4. To assess their quantity and size, both types of EVs were subjected to NTA. The numbers of mBALF-EVs and hBALF-EVs were significantly greater than those in the Sham group and the Non-ARDS group, respectively (Fig. 1A, B). The round morphology and size of the EVs were confirmed by TEM (Fig. 1C). WB analysis revealed the expression of characteristic markers of EVs, including the tetraspanin CD63, tumour susceptibility gene 101 (TSG101), and ALIX [42], in both mBALF-EVs and hBALF-EVs (Fig. 1D). Bivariate dot plots were generated to depict the relationship between FITC fluorescence and side scatter (SS-A) for mBALF-EVs and hBALF-EVs (Fig. 1E). Interestingly, compared to those in the control samples, the proportions of EC-EVs were significantly increased among both mBALF-EVs and hBALF-EVs (Fig. 1F).

**Fig. 2:**
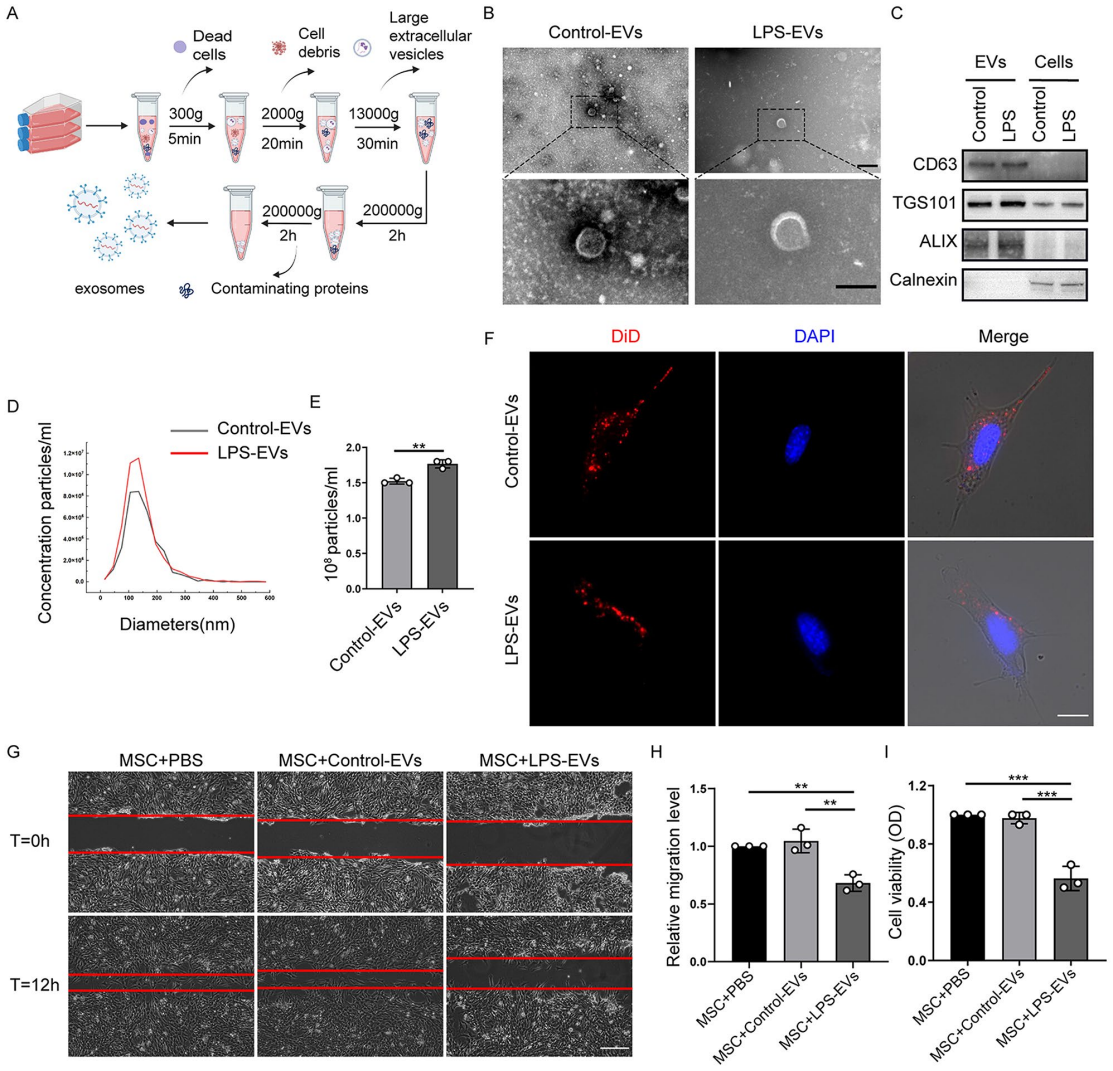
LPS-EVs attenuate the viability and migratory ability of BM-MSCs. **A** Schematic diagram of the isolation of EVs from LPS-untreated or treated pulmonary microvascular endothelial cells (Control-EVs or LPS-EVs). **B** TEM images of isolated Control-EVs and LPS-EVs. Scale bar: 200 nm (Top) and 100 nm (Bottom). **C** Immunoblot analysis of CD63, TSG 101, ALIX, and Calnexin in cell lysates and EV preparations. **D, E** NTA measurements illustrating the size distribution and concentration of Control-EVs and LPS-EVs. (*n* = 3) **F** Fluorescent staining image of Control-EVs and LPS-EVs taken up by BM-MSCs. Scale bar: 10 μm. The effect of EV treatment on BM-MSC migration was examined by in vitro scratch assay. The wound areas were photographed at 0 and 12 h **(G)** and quantified **(H)** by measuring the wound area in each group. (*n* = 3). Scale bar: 200 μm. **I** Cell viability of BM-MSCs incubated with Control-EVs or LPS-EVs was measured using the CCK-8 assay. (*n* = 3). Data are presented as mean ± SD using one-way ANOVA followed by the Tukey’s multiple comparisons test or unpaired *t* test. **P < 0.01, ***P < 0.001

We examined the changes in EC-EVs in the lung microenvironment of ALI mice and ARDS patients and found varying degrees of change. We speculated that the changes in injured EC-EVs may contribute to the unsatisfactory clinical efficacy of MSCs. Therefore, to investigate the effect of EC-EVs on MSCs in vitro, iMPMEC-EVs were isolated from the culture supernatants of LPS- or PBS-pretreated iMPMECs by differential ultracentrifugation (Fig. 2A). TEM revealed that both Control-EVs and LPS-EVs exhibited a cup-shaped morphology, which is typical of vesicles isolated by this ultracentrifugation technique (Fig. 2B). WB analysis demonstrated that iMPMEC-EVs expressed the EV protein markers CD63, TSG101, and ALIX but lacked the endoplasmic reticulum marker Calnexin (Fig. 2C). NTA also revealed that iMPMEC-EVs had diameters ranging from 100 to 300 nm (Fig. 2D) and that EV productions significantly increased after LPS stimulation (Fig. 2E). Next, we labelled iMPMEC-EVs with DiD, a lipophilic fluorescent dye commonly used to label cell membranes and other hydrophobic structures. After incubation with BM-MSCs, both Control-EVs and LPS-EVs were taken up by the BM-MSCs (Fig. 2F, Supplementary Fig. S2A). However, the dynamic uptake assay revealed no significant difference in the ability of BM-MSCs to take up Control-EVs or LPS-EVs (Supplementary Fig. S2B). Subsequently, we investigated the impact of iMPMEC-EVs on the viability and migration of BM-MSCs. The migration of BM-MSCs treated with LPS-EVs was significantly weaker than that of BM-MSCs treated with Control-EVs (Fig. 2G, H). Cell viability assays revealed decreased cell viability in the LPS-EV-treated BM-MSCs compared to the Control-EV-treated BM-MSCs (Fig. 2I). Additionally, we used murine lung epithelial-12 cells (MLE-12) as controls and found that LPS-EVs did not inhibit the cell viability and migration of MLE-12, ruling out a general toxic effect of LPS-EVs (Supplementary Fig. S3). We also eliminated the impact of residual LPS on MSC function by detecting the LPS concentrations in EV samples (Supplementary Table 5). These findings indicate that EV productions significantly increased under pathological conditions, and subsequent in vitro experiments demonstrated their capability to suppress the viability and migration of BM-MSCs.

### LPS-EVs weaken the therapeutic effects of BM-MSCs in ALI mice

**Fig. 3:**
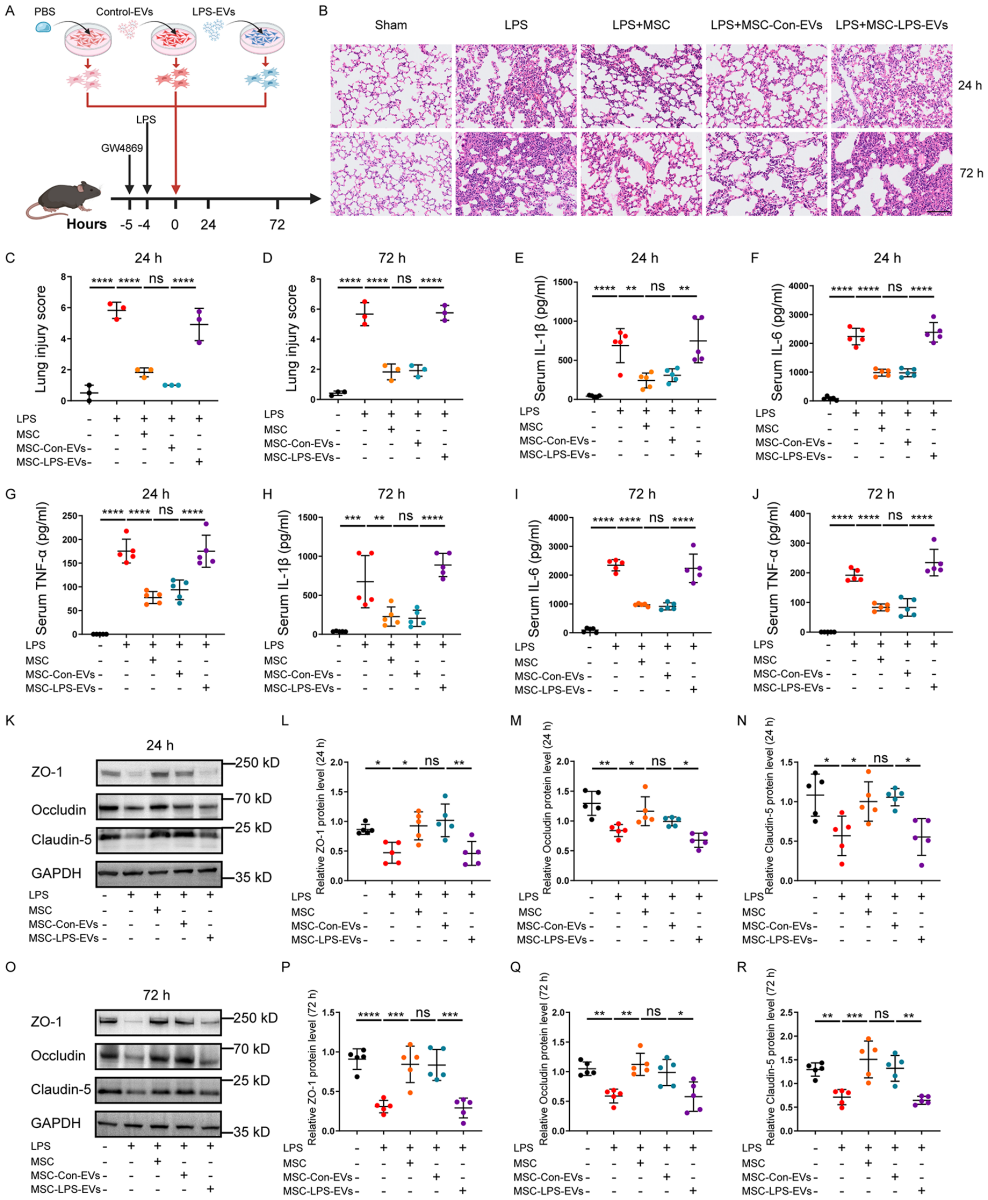
Pre-treatment of BM-MSCs with LPS-EVs abolishes the therapeutic effect of BM-MSCs in ALI mice. **A** Schematic protocol for BM-MSCs treatment in ALI mice. BM-MSCs were pretreated with or without Control-EVs or LPS-EVs in vitro for 24 h. **B** H&E staining of lung tissue sections of C57/BL6 mice at 24 and 72 h after BM-MSCs treatment. Scale bar: 100 μm. **C** Quantitative analysis of the lung injury scores at 24 h after BM-MSCs treatment. (*n* = 3). **D** Quantitative analysis of the lung injury scores at 72 h after BM-MSCs treatment. (*n* = 3). **E-G** Quantification of IL-1β, IL-6 and TNF-α concentrations in serum of mice 24 h after BM-MSCs treatment in LPS-induced ALI mice, measured using ELISA. (*n* = 5). **H-J** Quantification of IL-1β, IL-6 and TNF-α concentrations in serum of mice 72 h after BM-MSCs treatment in LPS-induced ALI mice, measured using ELISA. (*n* = 5). Representative western blot **(K)** and quantitative analysis of ZO-1 **(L)**, Occludin **(M)**, and Claudin-5 **(N)** in lung tissue in mice at 24 h post BM-MSCs administration in LPS-induced ALI. (*n* = 5). Representative western blot **(O)** and quantitative analysis of ZO-1 **(P)**, Occludin **(Q)**, and Claudin-5 **(R)** in lung tissue in mice at 72 h post BM-MSCs administration in LPS-induced ALI. (*n* = 5). Data are presented as mean ± SD using one-way ANOVA followed by the Tukey’s multiple comparisons test. *P < 0.05, **P < 0.01, ***P < 0.001, ****P < 0.0001

Cell viability and migration are essential properties of MSCs for carrying out tissue repair functions [43, 44]. . We observed that LPS-EVs reduced the viability and migration of BM-MSCs in vitro. To further explore the effect of LPS-EVs on the therapeutic efficacy of BM-MSCs in ALI mice, we conducted in vivo experiments (as depicted in Fig. 3A). The degree of lung injury was evaluated based on lung histology. Increased alveolar and interstitial inflammatory cell infiltration, thickening of the alveolar walls, and diffuse alveolar oedema, which result in a higher lung injury score, were evident in the LPS group and were reversed at both 24 and 72 h after the administration of BM-MSCs. There was no significant difference between the LPS + MSC group and the LPS + MSC-Con-EVs group. However, the histopathological characteristics were more severe and corresponding lung injury scores were significantly higher in the LPS + MSC-LPS-EVs group than in the LPS + MSC-Con-EVs group (Fig. 3B-D). These results suggest that MSCs have a protective effect on lung injury, but this effect is diminished when MSCs are pretreated with LPS-EVs.

We also assayed the levels of IL-1β, IL-6 and TNF-α in the serum of mice after treatment with BM-MSCs. LPS induced lung inflammation, as evidenced by increased IL-1β, IL-6 and TNF-α levels in the serum compared to those in the Sham group. After treatment with BM-MSCs pretreated with PBS or Control-EVs, the serum levels of IL-1β, IL-6 and TNF-α were significantly decreased, and these decreases were reversed by treatment with BM-MSCs pretreated with LPS-EVs at 24 and 72 h after MSC treatment (Fig. 3E-J). Similarly, RT‒qPCR analysis revealed that the relative *Il1b*, *Il6* and *Tnf* gene expression in the lungs of the LPS + MSC-LPS-EVs group was significantly greater at 24 h than that in the LPS + MSC-Con-EVs group (Supplementary Fig. S4A-C).

Stimulation of mouse lungs with LPS can increase pulmonary microvascular permeability through a process associated with tight junction proteins such as Occludin, Claudin-5, and zonula occluden-1 (ZO-1), leading to pulmonary oedema in mice [45, 46]. Pulmonary oedema can be quantified by calculating the ratio of LWW/BW. In this study, we investigated the impact of BM-MSC treatment on tight junction proteins and the LWW/BW ratio in lung tissues. WB analysis revealed a significant reduction in the levels of Occludin, Claudin-5, and ZO-1 at both 24 and 72 h after LPS-induced lung injury. Notably, the expression levels of all three tight junction proteins increased significantly in the LPS + MSC group, with no significant difference observed between the LPS + MSC group and the LPS + MSC-Con-EVs group. However, the expression of all three tight junction proteins was significantly lower in the LPS + MSC-LPS-EVs group than in the LPS + MSC-Con-EVs group at both 24 and 72 h after MSC treatment (Fig. 3K-R). Furthermore, the LWW/BW ratio significantly increased in the LPS group at both 24 and 72 h. After the administration of BM-MSCs, the LWW/BW ratio was significantly reduced, and this reduction was significantly reversed by the administration of BM-MSCs pretreated with LPS-EVs but not with Control-EVs at both 24 and 72 h after MSC treatment (Supplementary Fig. S4D, E). Collectively, the engraftment of BM-MSCs pretreated with LPS-EVs exhibited attenuated anti-inflammatory effects and pulmonary microvascular protection in mice with ALI.

### LPS-EVs inhibit the therapeutic effects of BM-MSCs by suppressing TET activity and mediating DNA hydroxymethylation

**Fig. 4:**
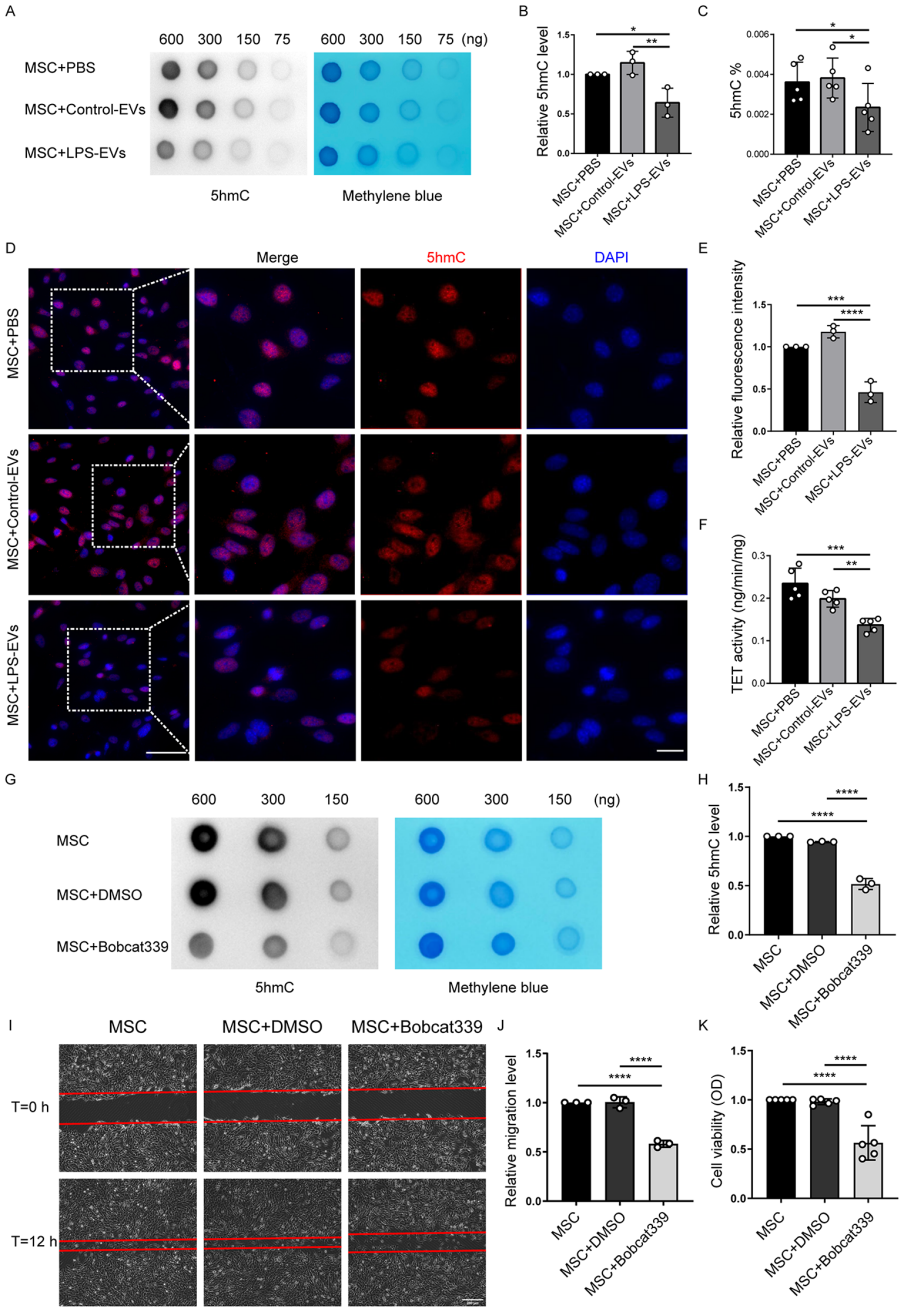
LPS-EVs suppress MSC viability and migratory ability by inhibiting DNA hydroxymethylation level and TET activity. A representative dot-blot **(A)** and semi-quantitative analysis **(B)** showed the content of 5hmC in BM-MSCs pretreated with Control-EVs or LPS-EVs for 24 h. (*n* = 3). **C** Global DNA 5hmC levels in the BM-MSCs were measured using the MethylFlash™ Hydroxymethylated DNA Quantification Kit (Fluorometric) (*n* = 5). **D, E** Representative images of immunofluorescence staining and relative fluorescence intensity analysis revealed the expression of 5hmC in BM-MSCs incubated with Control-EVs or LPS-EVs for 24 h. (*n* = 3). Scale bar: 50 μm (left) and 5 μm (right). **F** TET activity in BM-MSCs was assayed by the Epigenase™ 5mC Hydroxylase TET Activity/Inhibition Assay Kit (Fluorometric). (*n* = 5). A representative dot-blot **(G)** and semi-quantitative analysis **(H)** showed the content of 5hmC in BM-MSCs pretreated with TET enzyme inhibitor, Bobcat339. (*n* = 3). The effect of Bobcat339 treatment on BM-MSC migration was examined by in vitro scratch assay. The wound areas were photographed at 0 and 12 h **(I)** and quantified **(J)** by measuring the wound area in each group. (*n* = 3). Scale bar: 200 μm. **K** Cell viability of BM-MSCs incubated with Bobcat339 was measured using the CCK-8 assay. (*n* = 5). Data are presented as mean ± SD using one-way ANOVA followed by the Tukey’s multiple comparisons test. *P < 0.05, **P < 0.01, ***P < 0.001, ****P < 0.0001

A previous study suggested that epigenetic regulation, especially DNA methylation, plays a crucial role in MSC dysfunction [21]. TET, a key enzyme involved in DNA demethylation, catalyses the conversion of 5mC to 5hmC [27]. Thus, exploring the regulation of DNA hydroxymethylation in MSCs will provide deep insights into cell transplantation-based therapeutics for ARDS. In this study, DNA dot blot analysis revealed a significant reduction in 5hmC in BM-MSCs after 24 h of incubation with LPS-EVs compared to after incubation with PBS or Control-EVs (Fig. 4A, B). The global 5hmC content in DNA was assessed (Fig. 4C), and the results were consistent with the dot-blot analysis. Immunofluorescence staining further revealed a marked reduction in 5hmC mainly in the cell nuclei of BM-MSCs after treatment with LPS-EVs but not in those of Control-EVs (Fig. 4D, E). Notably, TET plays a key role in DNA hydroxymethylation [27]. We found that the three TET isoforms (TET 1–3) were expressed in BM-MSCs (Supplementary Fig. S5A). Changes in the expression levels of all three TET isoforms in BM-MSCs treated with LPS-EVs were not significant than in those treated with Control-EVs at either the protein level or the mRNA level, as assessed by Western blotting and RT‒qPCR, respectively (Supplementary Fig. S5B‒E). However, TET activity was significantly lower in BM-MSCs treated with LPS-EVs than in those treated with PBS or Control-EVs (Fig. 4F).

Furthermore, the TET enzyme inhibitor Bobcat339 was used to investigate the effect of TET activity and its ability to mediate DNA hydroxymethylation on the viability and migration of BM-MSCs. The results showed that Bobcat339 effectively reduced the level of 5hmC in BM-MSCs (Fig. 4G, H), and compared with DMSO, Bobcat339 significantly decreased cell viability and migration (Fig. 4I-K). Taken together, these findings demonstrate that LPS-EVs attenuate the viability and migration of BM-MSCs by downregulating TET activity and subsequently decreasing DNA hydroxymethylation.

### *Idh2* is the key molecule by which LPS-EVs inhibit TET activity and thereby impair the therapeutic effects of BM-MSCs

**Fig. 5:**
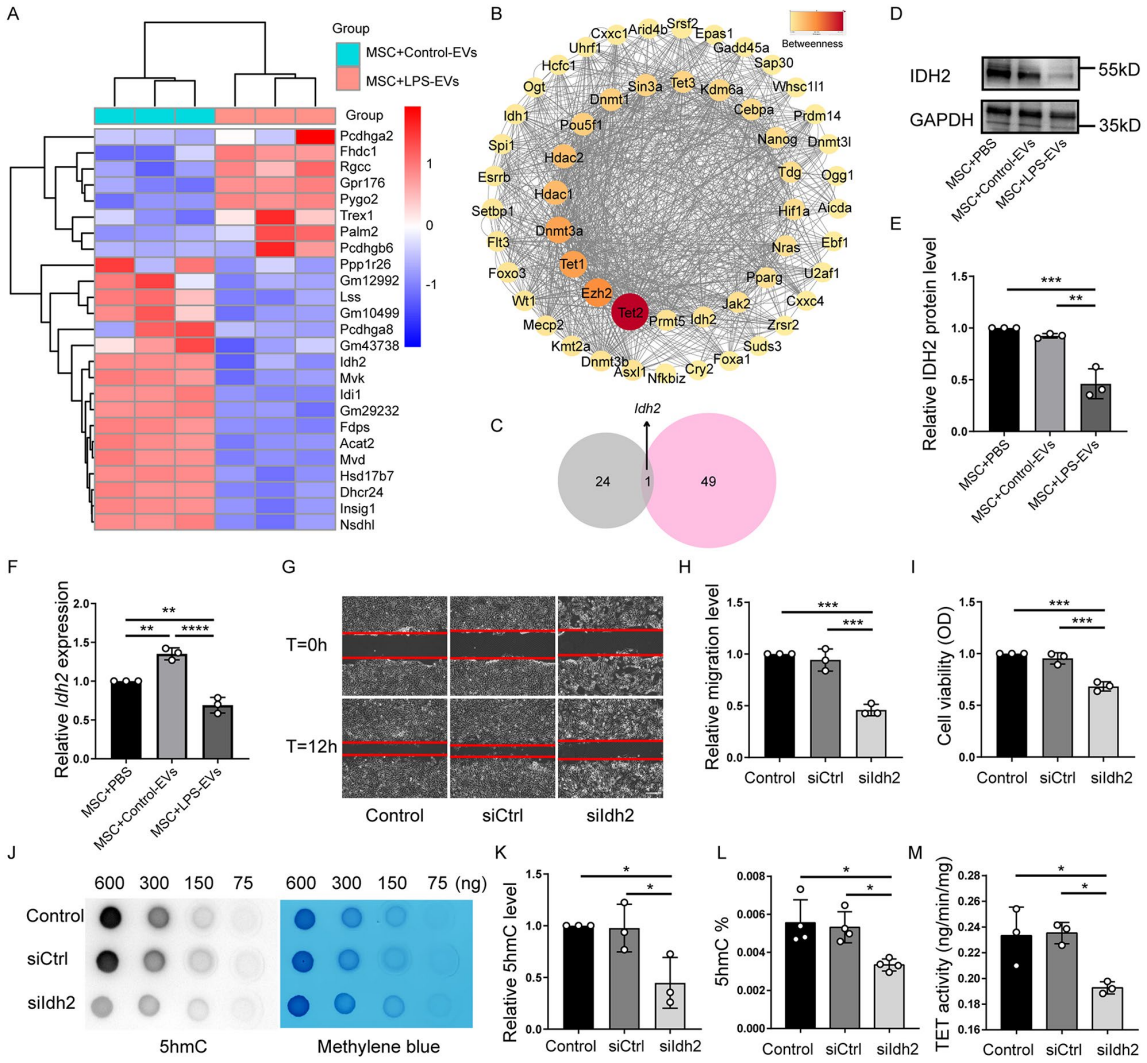
*Idh2* mediates the suppression of TET activity and the consequential inhibition in viability and migration of BM-MSCs by LPS-EVs. **A** Heat map displaying the differentially expressed genes (DEGs) in BM-MSCs after incubation with Control-EVs or LPS-EVs for 24 h. (*n* = 3). **B** The top 50 proteins that interact with Tet1, Tet2 and Tet3 were revealed using the String Protein Interaction Network (https://string-db.org). **C** Venn Diagram showing the intersection of the 25 DEGs between Control-EVs and LPS-EVs treated groups, and the 50 genes corresponding to the 50 proteins interacting with Tet1, Tet2, or Tet3. Representative western blot **(D)** and quantitative data **(E)** of IDH2 in BM-MSCs incubated with Control-EVs or LPS-EVs for 24 h. (*n* = 3). **F** Relative expression of *Idh2* in BM-MSCs incubated with Control-EVs or LPS-EVs for 24 h. (*n* = 3). Representative images **(G)** and quantitative analysis **(H)** of the migratory ability of BM-MSCs after treatment with siCtrl or siIdh2 were examined by in vitro scratch assay. (*n* = 3). Scale bar: 100 μm. **I** Cell viability of BM-MSCs incubated with siCtrl or siIdh2 was measured by CCK-8 assay. (*n* = 3). A representative dot-blot **(J)** and semi-quantitative analysis **(K)** showed the content of 5hmC in BM-MSCs incubated with siCtrl or siIdh2. **L** Global DNA 5hmC level in BM-MSCs incubated with siCtrl or siIdh2 was assayed by the MethylFlash™ Hydroxymethylated DNA Quantification Kit (Fluorometric). (*n* = 4). **M** TET activity in BM-MSCs incubated with siCtrl or siIdh2 was assayed by the Epigenase™ 5mC Hydroxylase TET Activity/Inhibition Assay Kit (Fluorometric). (*n* = 3). Data are presented as mean ± SD using one-way ANOVA followed by the Tukey’s multiple comparisons test. *P < 0.05, **P < 0.01, ***P < 0.001, ****P < 0.0001

To confirm the key factors that are affected by iMPMEC-EVs and contribute to the regulation of TET activity in BM-MSCs, RNA-seq was performed on BM-MSCs after treatment with Control-EVs or LPS-EVs. Compared to the Control-EV-treated group, 25 DEGs were identified, among which 8 DEGs were upregulated and 17 DEGs were downregulated, after treatment with LPS-EVs (Fig. 5A). To further explore the key genes regulating TET activity, 50 proteins that interact with TET1, TET2, or TET3 were identified using the STRING protein interaction network (Fig. 5B). The 25 DEGs between the Control-EV- and LPS-EV-treated groups and the 50 genes corresponding to the 50 proteins that interact with TET1, TET2, or TET3 were intersected. Interestingly, only one downregulated gene (isocitrate dehydrogenase 2, *Idh2*) was found to interact with the three TET proteins (Fig. 5C). Thus, we assessed the protein level of IDH2 in BM-MSCs after treatment with PBS or iMPMEC-EVs. The reduction in IDH2 protein level was significant in BM-MSCs after 24 h of incubation with LPS-EVs compared to incubation with PBS or Control-EVs (Fig. 5D, E). Furthermore, compared with that in the other groups, the mRNA level of *Idh2* in the BM-MSCs was significantly lower after treatment with LPS-EVs, as assessed by RT‒qPCR (Fig. 5F). Additionally, there was no difference in the expression of *Idh1* or *Idh3a* in BM-MSCs after treatment with PBS or iMPMEC-EVs (Supplementary Fig. S6A, B).

To further investigate the role of IDH2 in TET activity and TET-mediated DNA hydroxymethylation in BM-MSCs, siIdh2 was used to suppress the expression of *Idh2*. BM-MSCs transfected with siIdh2 for 48 h showed significant reductions in both protein and mRNA expression compared to that of cells transfected with siCtrl (Supplementary Fig. S7A-C). These results suggested successful silencing of *Idh2* in BM-MSCs. Furthermore, vacuolization was observed in BM-MSCs pretreated with siIdh2 (siIdh2-MSCs) (Supplementary Fig. S7H). Migration assay and CCK-8 assay revealed a significant reduction in migration and cell viability of BM-MSCs upon silencing of *Idh2*, respectively. (Fig. 5G-I). In addition, compared with those in the siCtrl group (siCtrl-MSCs), the global 5hmC level in the siIdh2 group was significantly lower (Fig. 4J-L), and the TET activity was correspondingly lower (Fig. 5M). Notably, there were no changes in the mRNA expression levels of any of the three *Tet* isoforms upon siIdh2 treatment (Supplementary Fig. S8A-C), suggesting that IDH2 modulates the activity of TET enzymes rather than their transcription. Our findings reveal the role of IDH2 in modulating TET activity and global DNA hydroxymethylation in BM-MSCs.

**Fig. 6:**
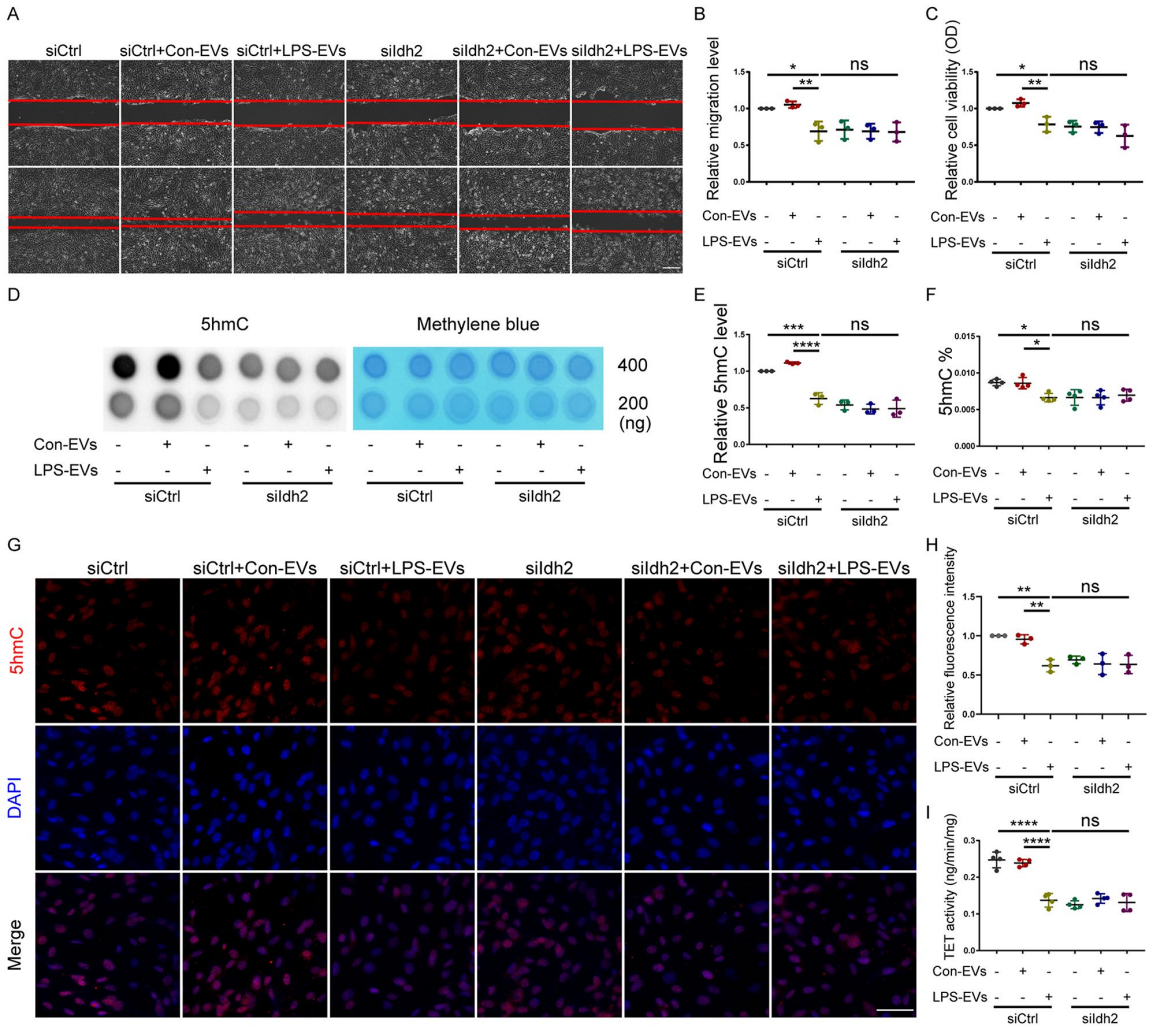
Knockdown of *Idh2* in BM-MSCs does not further aggravate the inhibitory effect of LPS-EVs on BM-MSCs. Representative images **(A)** and quantitative analysis **(B)** of the migratory ability of siCtrl and siIdh2 groups after treatment with Control-EVs or LPS-EVs were examined by in vitro scratch assay at 0 and 12 h. (*n* = 3). Scale bar: 100 μm. **C** Cell viability of siCtrl and siIdh2 groups after incubation with Control-EVs or LPS-EVs was measured by CCK-8 assay. (*n* = 3). A representative dot-blot **(D)** and semi-quantitative analysis **(E)** showed the content of 5hmC in siCtrl and siIdh2 groups after incubation with Control-EVs or LPS-EVs (*n* = 3). **F** Global DNA 5hmC levels in siCtrl and siIdh2 groups after incubation with Control-EVs or LPS-EVs were assayed by the MethylFlash™ Hydroxymethylated DNA Quantification Kit (Fluorometric). (*n* = 4). Representative images of immunofluorescence staining **(G)** and relative fluorescence intensity analysis **(H)** demonstrated the expression of 5hmC in siCtrl and siIdh2 groups after incubation with Control-EVs or LPS-EVs for 24 h. (*n* = 3) Scale bar: 50 μm. **I** TET activity in siCtrl and siIdh2 groups after incubation with Control-EVs or LPS-EVs was assayed by the Epigenase™ 5mC Hydroxylase TET Activity/Inhibition Assay Kit (Fluorometric). (*n* = 4). Data are presented as mean ± SD using one-way ANOVA followed by the Tukey’s multiple comparisons test. *P < 0.05, **P < 0.01, ***P < 0.001,****P < 0.0001.

To determine whether LPS-EVs could further inhibit the viability and migratory ability of siIdh2-MSCs through an epigenetic pathway, siIdh2-MSCs were cocultured with LPS-EVs. Our data suggested that knockdown of *Idh2* in BM-MSCs did not further exacerbate the inhibitory effect of LPS-EVs on BM-MSC viability or migration (Fig. 6A-C). Moreover, compared with siCtrl-MSCs treated with LPS-EVs, siIdh2-MSCs treated with LPS-EVs did not exhibit significantly reduced global 5hmC content, as determined by dot blotting, the MethylFlash™ Hydroxymethylated DNA Quantification Kit, or immunofluorescence staining (Fig. 6D-H). Furthermore, TET activity did not significantly decrease in siIdh2-MSCs treated with LPS-EVs compared to that in siCtrl-MSCs treated with LPS-EVs (Fig. 6I).

### Overexpression of *Idh2* rescues the decrease in cell viability and migration observed in BM-MSCs after LPS-EV intervention through an epigenetic pathway

**Fig. 7:**
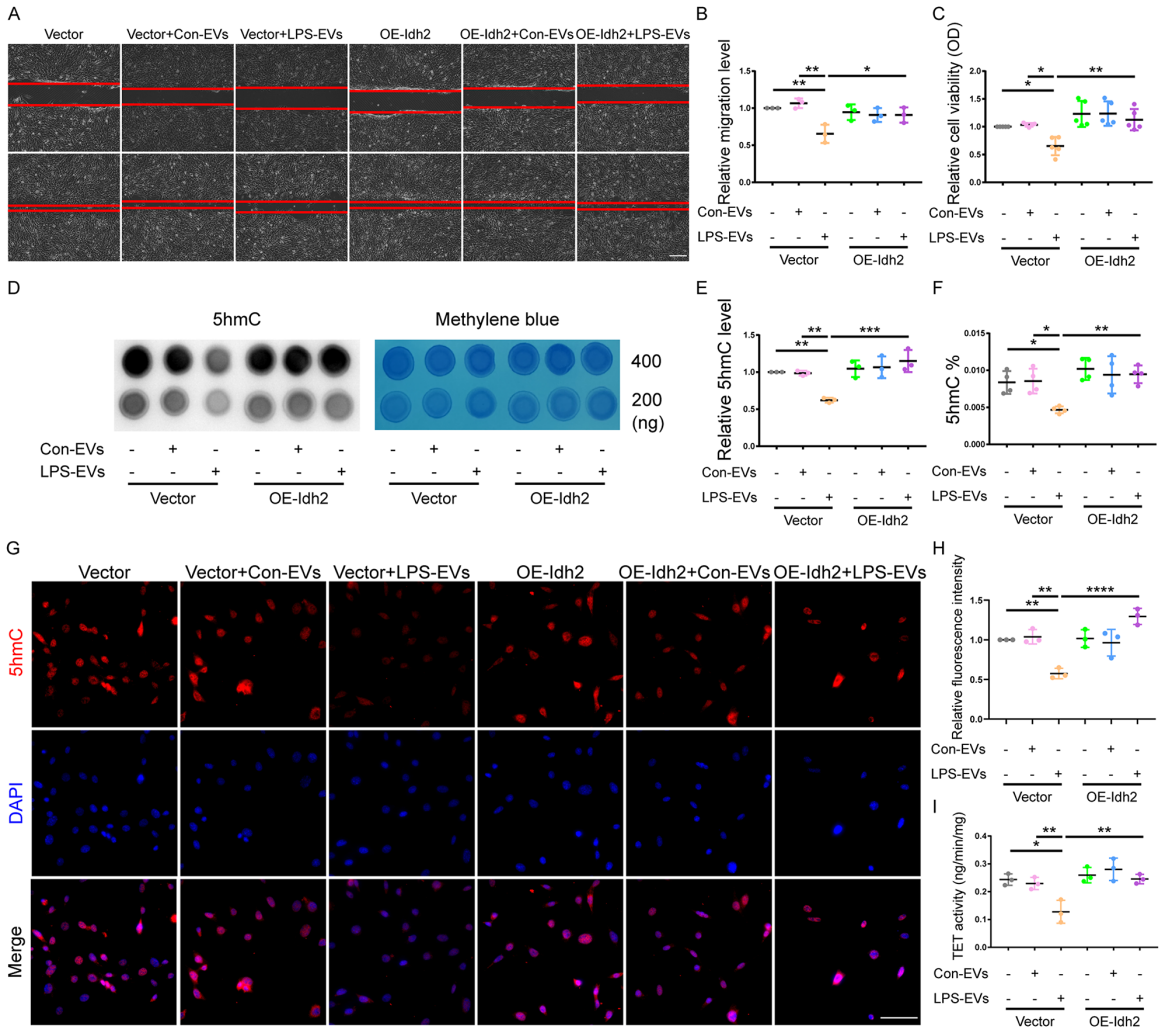
Overexpression of *Idh2* in BM-MSCs reverses the inhibitory effects of LPS-EVs on BM-MSCs. Representative images **(A)** and quantitative analysis **(B)** of the migratory ability of Vector-MSCs and OE-Idh2-MSCs after treated with Control-EVs or LPS-EVs, evaluated using an in vitro scratch assay at 0 and 12 h. (*n* = 3). Scale bar: 100 μm. **C** Cell viability of Vector-MSCs and OE-Idh2-MSCs incubated with Control-EVs or LPS-EVs was measured by CCK-8 assay. (*n* = 5). A representative dot-blot **(D)** and semi-quantitative analysis **(E)** showed the content of 5hmC in Vector-MSCs and OE-Idh2-MSCs after incubation with Control-EVs or LPS-EVs. (*n* = 3). **F** Global DNA 5hmC level in Vector-MSCs and OE-Idh2-MSCs after incubation with Control-EVs or LPS-EVs was assayed by the MethylFlash™ Hydroxymethylated DNA Quantification Kit (Fluorometric). (*n* = 4). Representative images of immunofluorescence staining **(G)** and relative fluorescence intensity analysis **(H)** demonstrated the expression of 5hmC in Vector-MSCs and OE-Idh2-MSCs after incubation with Control-EVs or LPS-EVs for 24 h. (*n* = 3). Scale bar: 50 μm. **I** TET activity in Vector-MSCs and OE-Idh2-MSCs after incubation with Control-EVs or LPS-EVs was assayed by the Epigenase™ 5mC Hydroxylase TET Activity/Inhibition Assay Kit (Fluorometric). (*n* = 3). Data are presented as mean ± SD using one-way ANOVA followed by the Tukey’s multiple comparisons test. *P < 0.05, **P < 0.01, ***P < 0.001, ****P < 0.0001

First, we investigated whether the overexpression of *Idh2* in BM-MSCs (OE-Idh2-MSCs) cocultured with LPS-EVs could reverse the reduction in TET activity and DNA hydroxymethylation caused by LPS-EVs. Our data suggested that, compared with Vector-MSCs, OE-Idh2-MSCs significantly increased both the mRNA and protein expression of IDH2 (Supplementary Fig. S7D-G). Overexpression of *Idh2* had no effect on the morphology of the BM-MSCs (Supplementary Fig. S7H). Furthermore, Migration assay and CCK-8 assay demonstrated that overexpression of *Idh2* significantly reversed the decreases in migration and cell viability of BM-MSCs cocultured with LPS-EVs, respectively (Fig. 7A-C). Moreover, compared with Vector-MSCs treated with LPS-EVs, OE-Idh2-MSCs treated with LPS-EVs had significantly higher global 5hmC levels according to Dot-blot, MethylFlash™ Hydroxymethylated DNA Quantification Kit, and immunofluorescence staining (Fig. 7D-H). Furthermore, TET activity in OE-Idh2-MSCs treated with LPS-EVs was significantly greater than that in Vector-MSCs treated with LPS-EVs (Fig. 7I).

### Overexpression of *Idh2* enhances the therapeutic effects of BM-MSCs in ALI mice

**Fig. 8:**
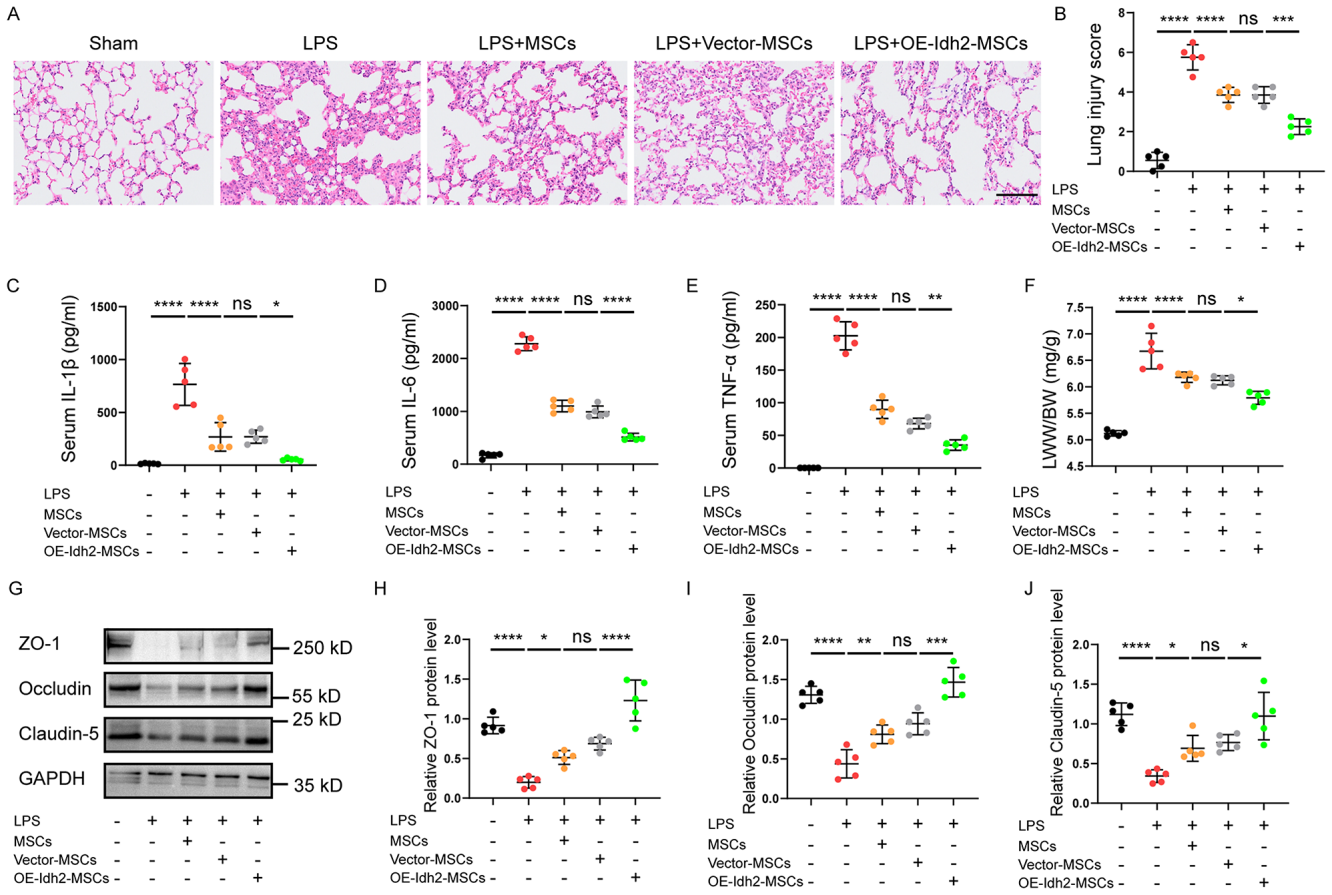
Overexpression of *Idh2* enhances the therapeutic effects of BM-MSCs in LPS-induced ALI. **A** H&E staining of lung tissue sections of C57/BL6 mice at 24 h after BM-MSCs treatment. Scale bar: 100 μm. (*n* = 5). **B** Quantitative analysis of the lung injury scores in each group at 24 h after BM-MSCs treatment. (*n* = 5). **C-E** Quantification of IL-1β, IL-6 and TNF-α concentrations in serum of mice at 24 h after BM-MSCs treatment in LPS-induced ALI mice, measured using ELISA. (*n* = 5). **F** The ratios of LWW/BW at 24 h post BM-MSCs treatment in the lung tissue of ALI mice. (*n* = 5). Representative western blot **(G)** and quantitative analysis of ZO-1 **(H)**, Occludin **(I)**, and Claudin-5 **(J)** in the lung tissue in mice at 24 h post BM-MSCs administration in LPS-induced ALI. (*n* = 5). Data are presented as mean ± SD using one-way ANOVA followed by the Tukey’s multiple comparisons test. *P < 0.05, **P < 0.01, ***P < 0.001, ****P < 0.0001

In order to elucidate the impact of *Idh2* overexpression on the reparative function of BM-MSCs, we employed BM-MSCs overexpressing *Idh2* for the treatment of ALI mice. The degree of lung injury was evaluated based on lung histology. The increase in lung injury in the LPS group was reversed after the administration of BM-MSCs. In addition, there was no significant difference between the LPS + MSCs group and the LPS + Vector-MSCs group. However, the histopathological characteristics were less severe and the corresponding lung injury scores were significantly lower in the LPS + OE-Idh2-MSCs group than in the LPS + Vector-MSCs group (Fig. 8A, B). These results suggest that the overexpression of *Idh2* in BM-MSCs has a protective effect against lung injury.

We also assayed the levels of IL-1β, IL-6 and TNF-α in the serum of mice after treatment with BM-MSCs. LPS induced lung inflammation, as evidenced by increased IL-1β, IL-6 and TNF-α levels in the serum compared to those in the Sham group. After BM-MSC treatment, both the serum IL-1β, IL-6 and TNF-α levels were significantly decreased, and the inflammatory cytokine levels were further decreased by treatment with OE-Idh2-MSCs (Fig. 8C-E). We also investigated the impacts of various BM-MSC treatments on tight junction proteins and the LWW/BW ratio in lung tissues. The LWW/BW ratio was significantly higher in the LPS group than in the Sham group but was significantly lower after the administration of BM-MSCs, and the LWW/BW ratio was also significantly lower after the administration of OE-Idh2-MSCs than after the administration of Vector-MSCs (Fig. 8F). WB analysis revealed significant reductions in the levels of Occludin, Claudin-5, and ZO-1 after LPS-induced lung injury. Notably, the expression levels of all three of these tight junction proteins increased significantly in the LPS + MSCs group, with no significant difference observed between the LPS + MSCs group and the LPS + Vector-MSCs group. However, the expression levels of all three tight junction proteins were significantly greater in the LPS + OE-Idh2-MSCs group than in the LPS + Vector-MSCs group (Fig. 8G-J). Taken together, these findings indicate that the overexpression of *Idh2* in BM-MSCs can enhance the reparative effects of these cells in ALI mice.

### LPS-EVs reduce the production of α-KG by inhibiting *Idh2* expression

**Fig. 9:**
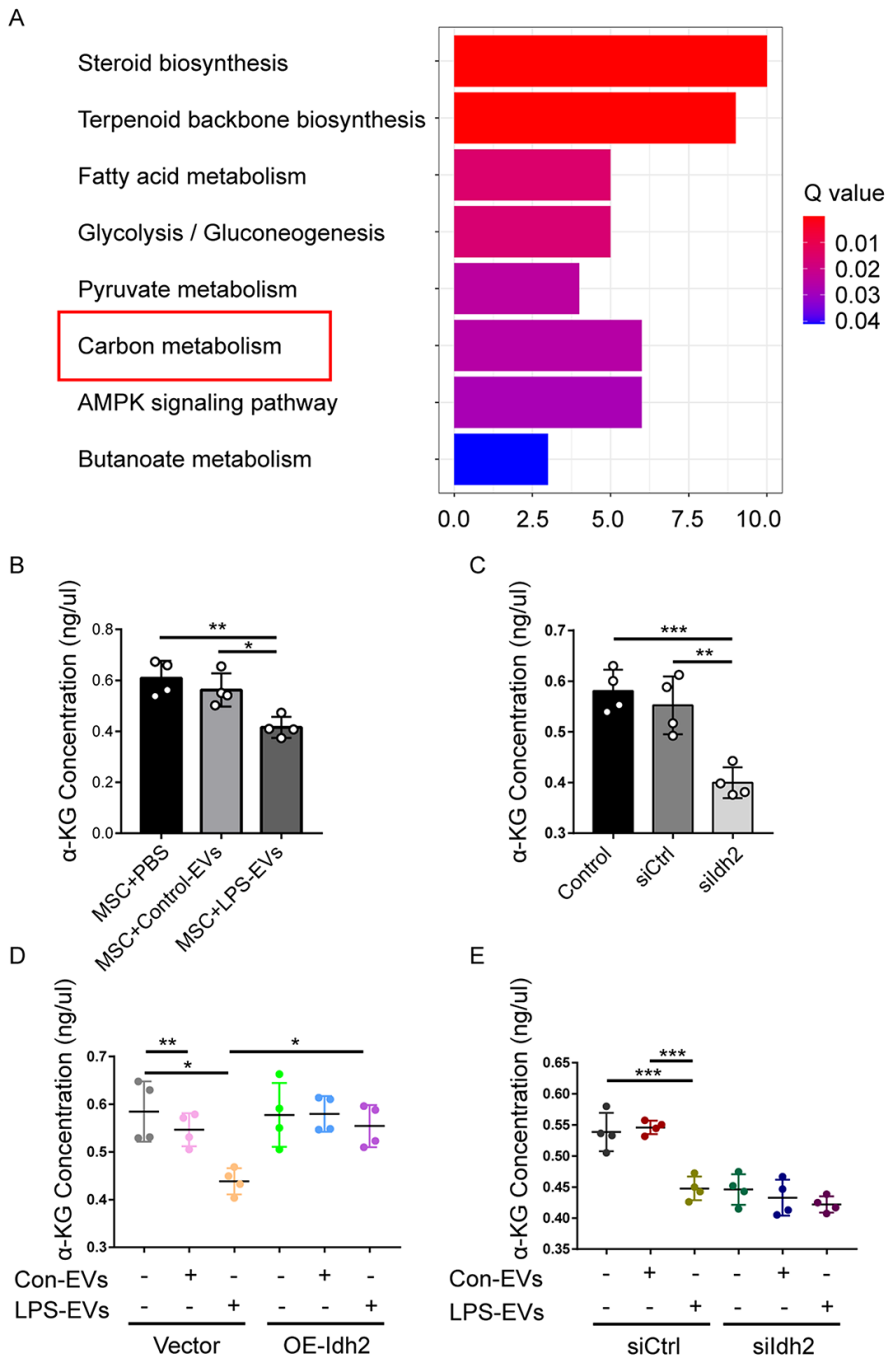
LPS-EVs reduce the production of α-KG, a key cofactor of TET activity, by inhibiting *Idh2* expression. **A** DEGs between BM-MSCs pretreated with Control-EVs and LPS-EVs were enriched by a KEGG pathway analysis. **B** α-KG concentration in BM-MSCs pretreated with Control-EVs or LPS-EVs was measured using the α-KG Assay Kit. (*n* = 4). **C** α-KG concentration in BM-MSCs incubated with siCtrl or siIdh2 was measured using the α-KG Assay Kit. (*n* = 4). **D** α-KG concentration in Vector-MSCs and OE-Idh2-MSCs after incubation with Control-EVs or LPS-EVs was measured using the α-KG Assay Kit. (*n* = 4). **E** α-KG concentration in siCtrl and siIdh2 groups after incubation with Control-EVs or LPS-EVs was measured using the α-KG Assay Kit. (*n* = 4). Data are presented as mean ± SD using one-way ANOVA followed by the Tukey’s multiple comparisons test. *P < 0.05, **P < 0.01, ***P < 0.001

To explore how IDH2 influences TET activity, KEGG pathway analysis was conducted, which showed that the MSC genes with differential expression between the Control-EV- and LPS-EV-treated groups were enriched mainly in metabolic pathways (Fig. 9A); the downregulated gene *Idh2* plays a crucial role in the tricarboxylic acid cycle (TCA cycle, Krebs cycle), a carbon metabolism pathway (Supplementary Fig. S9 is available at https://www.kegg.jp/pathway/map01200). IDH2 catalyses the conversion of isocitrate to α-ketoglutarate (α-KG), which is an important cofactor for the TET enzyme [25]. Therefore, we investigated whether LPS-EVs modulate the production of α-KG by affecting the availability of IDH2. We found that the BM-MSCs treated with LPS-EVs exhibited significantly lower α-KG levels than did those treated with PBS or Control-EVs (Fig. 9B). Consistent with these findings, we observed a significant decrease in the α-KG level in the siIdh2 group compared to the siCtrl group (Fig. 9C). Furthermore, compared with Vector-MSCs treated with LPS-EVs, OE-Idh2-MSCs treated with LPS-EVs exhibited a significant increase in the α-KG level (Fig. 9D). However, knockdown of *Idh2* in BM-MSCs did not further decrease the α-KG level in BM-MSCs cocultured with LPS-EVs (Fig. 9E). Taken together, these findings demonstrated that LPS-EVs reduce the production of α-KG likely by inhibiting the expression of *Idh2*.

**Fig. 10:**
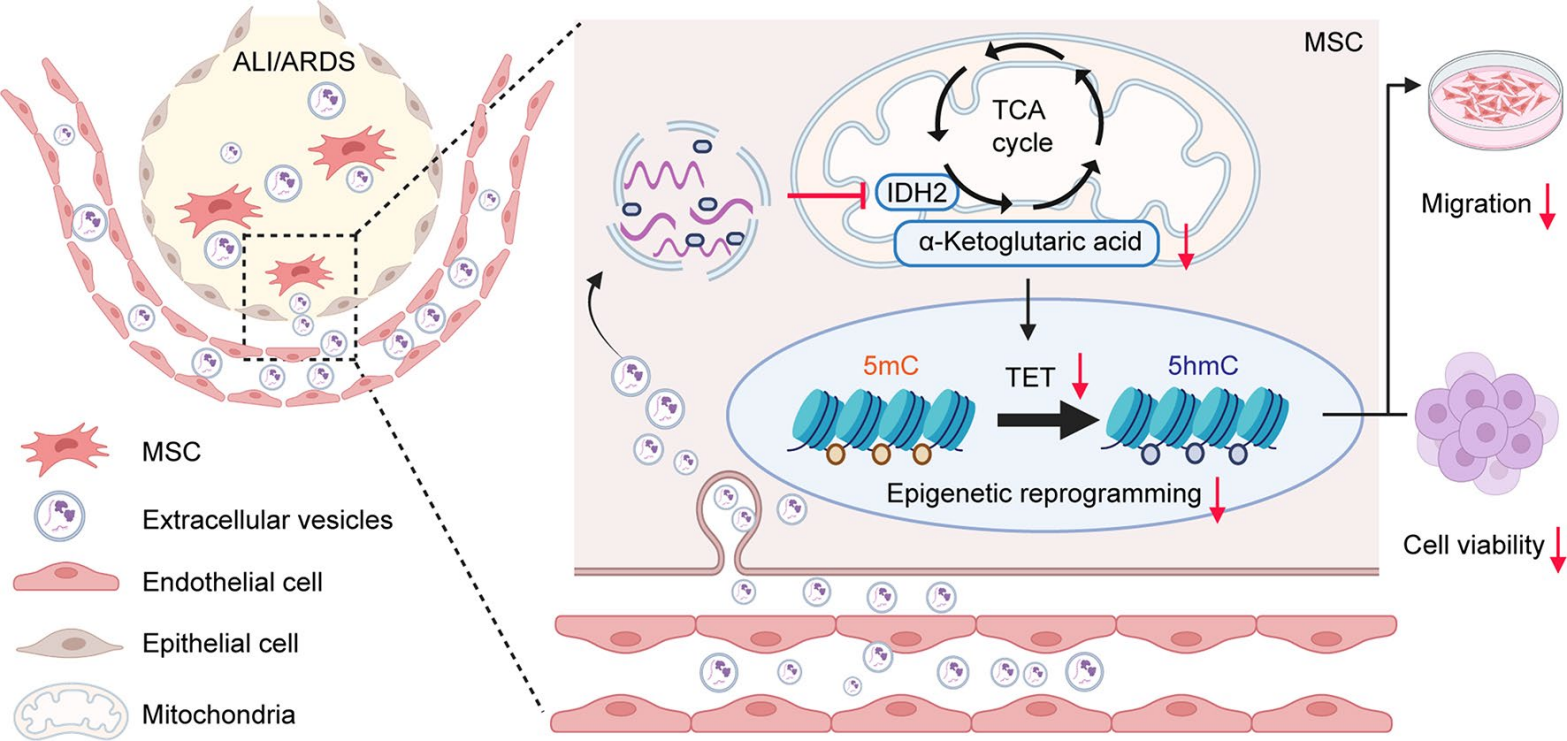
The proposed model for the role of LPS-EVs in modulating MSC functions through the metabolic-epigenic pathway

## Discussion

In our present study, we provide deep mechanistic insight into the effects of extracellular vesicles derived from pulmonary microvascular endothelial cells in experimental models of ARDS treated with BM-MSCs (Fig. 10). We found that compared with mice transplanted with unmanipulated BM-MSCs or BM-MSCs cultured with Control-EVs, the ALI mice receiving BM-MSCs cocultured with LPS-EVs exhibited significantly exacerbated pulmonary pathology, as well as increased inflammatory cytokine release and pulmonary capillary permeability. Subsequently, we reported that LPS-EVs reduced the viability and migration of BM-MSCs, likely by decreasing TET activity and TET-mediated hydroxymethylation. In addition, we provided novel evidence that the downregulation of TET activity might be modulated by IDH2, a protein involved in the citric acid cycle. Therefore, we explored the role of IDH2 in the regulation of TET activity in BM-MSCs. Our study demonstrated that overexpression of *Idh2* can reverse the inhibitory effects of LPS-EVs on MSC viability and migration through an epigenetic pathway. Furthermore, MSCs overexpressing *Idh2* had stronger protective effects on lung permeability and lung injury in vivo than did control MSCs.

As previously demonstrated, MSC treatment has shown substantial efficacy in animal models and preclinical studies of ALI due to its anti-inflammatory, antiapoptotic, and immunomodulatory effects [8, 9, 47, 48], but its efficacy in clinical investigations has been unsatisfactory [9]. An increasing number of studies have shown that the lung microenvironment can differentially influence various MSC behaviours [12–14]. The administration of MSCs protected the lung from ventilator-induced injury, whereas it worsened acid-primed lung injury. The lung proteome suggested a potential link between these effects and distinct proteomic profiles between the two groups [12]. Interestingly, other changes in the microenvironment, such as changes in oxygen partial pressure, substrate stiffness, and mechanical stretching [49–51], have been shown to affect MSC behaviours. In our study, EVs, another important components of the lung microenvironment, were found to regulate the cell viability and migration of MSCs. Previous studies have demonstrated the considerable therapeutic potential of MSCs in regenerative medicine, with their efficacy depending on cell viability and migratory ability to target tissues post-administration [43, 44]. However, MSC homing is notably inefficient, particularly in the lungs, where only about 5% of cells reach their destination, significantly limiting their therapeutic impact on lung damage [52, 53]. Addressing this issue, our previous study demonstrated that LincRNA-p21 enhances MSC migration under hypoxic conditions via the HIF-1α and CXCR4/7 pathways [54], making efforts to improve homing ability. Our current research has found that LPS-EVs can inhibit the migration of BM-MSCs, which reveals a critical mechanism behind the low MSC homing rates and has important clinical relevance.

Recently, accumulating evidence has suggested that EVs are important components of the inflammatory microenvironment in ALI/ARDS. Injured and dysfunctional cells often secrete EVs carrying altered cargoes into the serum or BALF [16, 55]. EVs are recognized as crucial mediators of intercellular communication and can regulate the activity of recipient cells by transporting cargoes such as lipids, proteins, and nucleic acids from their parent cells [15, 56, 57]. Moreover, pulmonary microvascular endothelial cell damage is the primary pathophysiological change in ARDS [58]. EVs released from injured endothelial cells can mediate inflammatory responses and have damaging effects [59–61]. This finding is consistent with our previous findings, which demonstrated that most EVs released from the perfusate in an ex vivo perfused lung model of bacterial pneumonia were secreted by endothelial cells and platelets and that these EVs can cause lung injury similar to that caused by *Escherichia coli* pneumonia [20]. However, whether EVs secreted by injured ECs affect the function of transplanted MSCs remains unclear. In this study, we found significant increases in the number of CD31-positive hBALF-EVs and the number of CD31-positive mBALF-EVs. In addition, the proportion of CD31-positive BALF-EVs in ARDS patients was significantly greater than that in ALI mice. A significant increase in the number of hBALF-EVs may affect the therapeutic efficacy of BM-MSCs, leading to unsatisfactory clinical outcomes. Therefore, we cultured iMPMECs, extracted a large quantity of iMPMEC-EVs, and cocultured these EVs with BM-MSCs in vitro prior to transplantation into ALI mice. We discovered that LPS-EVs attenuated the reparative effect of BM-MSCs on ALI. These results suggest a previously unidentified phenomenon that EC-EVs increased markedly in the lung microenvironment of ALI/ARDS, and these damaged EC-EVs can inhibit the therapeutic efficacy of transplanted MSCs on lung injury, which provides new insights into our understanding of the interaction between MSCs and the lung microenvironment.

Notably, lung-derived extracellular vesicles have been reported to be internalized by stem/progenitor cells, resulting in persistent gene and protein expression in pulmonary epithelial cells in vitro. Robust changes in cell fate are induced by lung-derived microvesicles in bone marrow cells [62–64]. Recently, epigenetic regulation, especially DNA methylation, has been shown to play a key role in MSC dysfunction [21]. However, whether EVs affect DNA methylation in MSCs remains unexplored. In our study, we observed a significant increase in the number of EVs isolated from iMPMECs after LPS stimulation, and these LPS-EVs were found to suppress global hydroxymethylation levels by inhibiting TET activity. Thus, we propose that EC-EVs influence BM-MSC function through an epigenetic pathway.

Previous reports have established that TET proteins are α-KG-dependent enzymes that facilitate the oxidation of 5mC to 5hmC. The production of α-KG is catalysed by isocitrate dehydrogenase (IDH) [26, 27, 65]. The IDH family contains three different isoforms: cytosolic NADP^+^-dependent IDH1, mitochondrial NADP^+^-dependent IDH2, and mitochondrial NAD^+^-dependent IDH3 [66]. In the present study, compared with Control-EVs, BM-MSCs presented a decrease in the α-KG level associated with reduced TET activity and global 5hmC levels after incubation with LPS-EVs. In addition, we provided evidence that the decrease in the α-KG level in LPS-EV-exposed BM-MSCs could be attributed to decreased expression of *Idh2*, which functions in the conversion of isocitrate in the citric acid cycle. We did not find any significant differences in the mRNA levels of cytosolic *Idh1* or another mitochondrial isoform, *Idh3a*, between BM-MSCs treated with LPS-EVs and those treated with Control-EVs, suggesting that the downregulation of *Idh2* may contribute to the observed decrease in the α-KG level in BM-MSCs. Therefore, siRNA was used to silence the expression of *Idh2*. Knockdown of *Idh2* altered the production of α-KG, subsequently impacting TET activity and 5hmC production due to the limited availability of the α-KG substrate. In addition, knockdown of *Idh2* reduced the viability and migratory ability of BM-MSCs, mimicking the inhibitory effects observed in LPS-EV-stimulated cells, further indicating that LPS-EV-mediated inhibition of the IDH2/TET pathway leads to decreased cell viability and migration in BM-MSCs. Thus, we demonstrated that overexpression of *Idh2* can reverse the inhibitory effects of LPS-EVs on the viability and migration of BM-MSCs through the IDH2/TET pathway. However, in unstimulated BM-MSCs, the overexpression of *Idh2* tended to increase the α-KG level and TET activity without causing significant changes in the global 5hmC level. We hypothesized that overexpression of *Idh2* may not upregulate the TCA cycle-driven production of α-KG or TET activity to a level that can induce significant epigenetic changes in normal cells under homeostatic conditions.

We found that EC-EVs in the pathological state could impair the reparative effect of BM-MSCs through the IDH2/TET pathway in vivo. We further investigated whether BM-MSCs overexpressing *Idh2* could reverse the inhibitory effects of pathological EC-EVs on BM-MSCs in vivo. Previous studies showed that the lung microenvironment during acute lung injury is inhabited by an extensive array of extracellular vesicles, originating from various cellular types and playing pivotal roles in the disease’s pathogenesis [16, 55]. To mimic the pathologically increased EC-EVs in vivo, we administered OE-Idh2-MSCs into the airways of ALI mice without GW4869 intervention. We found that OE-Idh2-MSCs alleviated lung injury in ALI mice. Our previous investigation revealed that EC-EVs constituted the predominant fraction in an ex vivo perfused human lung model and administration of these EVs resulted in ALI [20, 67]. So it is reasonable to suppose that EC-EVs are crucial in modulating the therapeutic effects of BM-MSCs. However, we cannot rule out the possibility that the EVs derived by other cells may also influence the therapeutic effects of MSCs.

Our study has some limitations. First, we have not yet identified the key molecules, which may be proteins or nucleic acids, within EVs that interact with IDH2 in BM-MSCs. Second, while we observed changes in the global hydroxymethylation level after treatment with EC-EVs, the specific changes at key sites remain unclear. Therefore, further investigations, such as proteomic or transcriptome profiling of the EVs, as well as methylation site sequencing of EV-pretreated MSCs, are needed to further explore these aspects.

## Conclusion

In conclusion, we demonstrated that EVs produced in a pathological state can inhibit the viability and migratory ability of BM-MSCs through a metabolic-epigenetic pathway, thereby attenuating the reparative effect of transplanted BM-MSCs on ALI, which provides new insights into the effect of the pulmonary microenvironment on the therapeutic efficacy of BM-MSCs. Furthermore, the development of treatment agents targeting the IDH2 will provide a potential strategy for improving the clinical therapeutic efficacy of MSC-based therapy.

### Electronic supplementary material

Below is the link to the electronic supplementary material.

Supplementary Material 1

Supplementary Material 2

Supplementary Material 3

## Data Availability

Data will be made available on request.

## Abbreviations

5caC: 5-carboxycytosine
5fC: 5-formylcytosine
5hmC: 5-hydroxymethylcytosine
5mC: 5-methylcytosine
α-KG: α-Ketoglutarate
ALI: Acute Lung Injury
ARDS: Acute Respiratory Distress Syndrome
BALF: Bronchoalveolar Lavage Fluid
BM-MSC: Bone Marrow Derived Mesenchymal Stem Cell
BSA: Bovine Serum Albumin
CCK−8: Cell Counting Kit−8
CCM: Conditioned Culture Medium
Control-EVs: Extracellular Vesicles Derived From Normal Endothelial Cells
EC-EVs: Endothelial Cell Derived Extracellular Vesicles
ECGS: Endothelial Cell Growth Supplement
EVs: Extracellular Vesicles
FBS: Fetal Bovine Serum
FGF−7: Fibroblast Growth Factor 7
hBALF-EVs: Extracellular Vesicles Isolated From Human Bronchoalveolar Lavage Fluid
hMSCs: Human Bone Marrow-Derived Mesenchymal Stem Cells
HRP: Horseradish Peroxidase
IDH: Isocitrate Dehydrogenase
IL: Interleukin
iMPMECs: Immortalized Mouse Pulmonary Microvascular Endothelial Cells
LPS: Lipopolysaccharide
LPS-EVs: Extracellular Vesicles Derived From Lps-Stimulated Endothelial Cells
LWW/BW: Lung Wet Weight/Body Weight
mBALF-EVs: Extracellular Vesicles Isolated From Mouse Bronchoalveolar Lavage Fluid
MSC: Mesenchymal Stem Cell
nFCM: Nano-Flow Cytometer
NTA: Nanoparticle Tracking Analysis
PVDF: Polyvinylidene Difluoride
qRT-PCR: Quantitative Real-Time Fluorescent Polymerase Chain Reaction
siRNA: Small Interfering RNA
TBS-T: Tris-Buffered Saline Containing 0.1% Tween-20
TCA: Tricarboxylic Acid Cycle
TEM: Transmission Electron Microscopy
TET: Ten-Eleven Translocation
TNF-α: Tumor Necrosis Factor α
TSG101: Tumor Susceptibility Gene 101
WB: Western Blot
