# Supplementary material for: Endothelial cell-derived extracellular vesicles modulate the therapeutic efficacy of mesenchymal stem cells through IDH2/TET pathway in ARDS
Source: Cell Commun Signal. 2024 May 27;22:293. doi: 10.1186/s12964-024-01672-0 (PMC11129421; doi:10.1186/s12964-024-01672-0)
Supplement: Supplementary file 2 — Supplementary Material 2 [file 12964_2024_1672_MOESM2_ESM.pdf]

Figure 1D

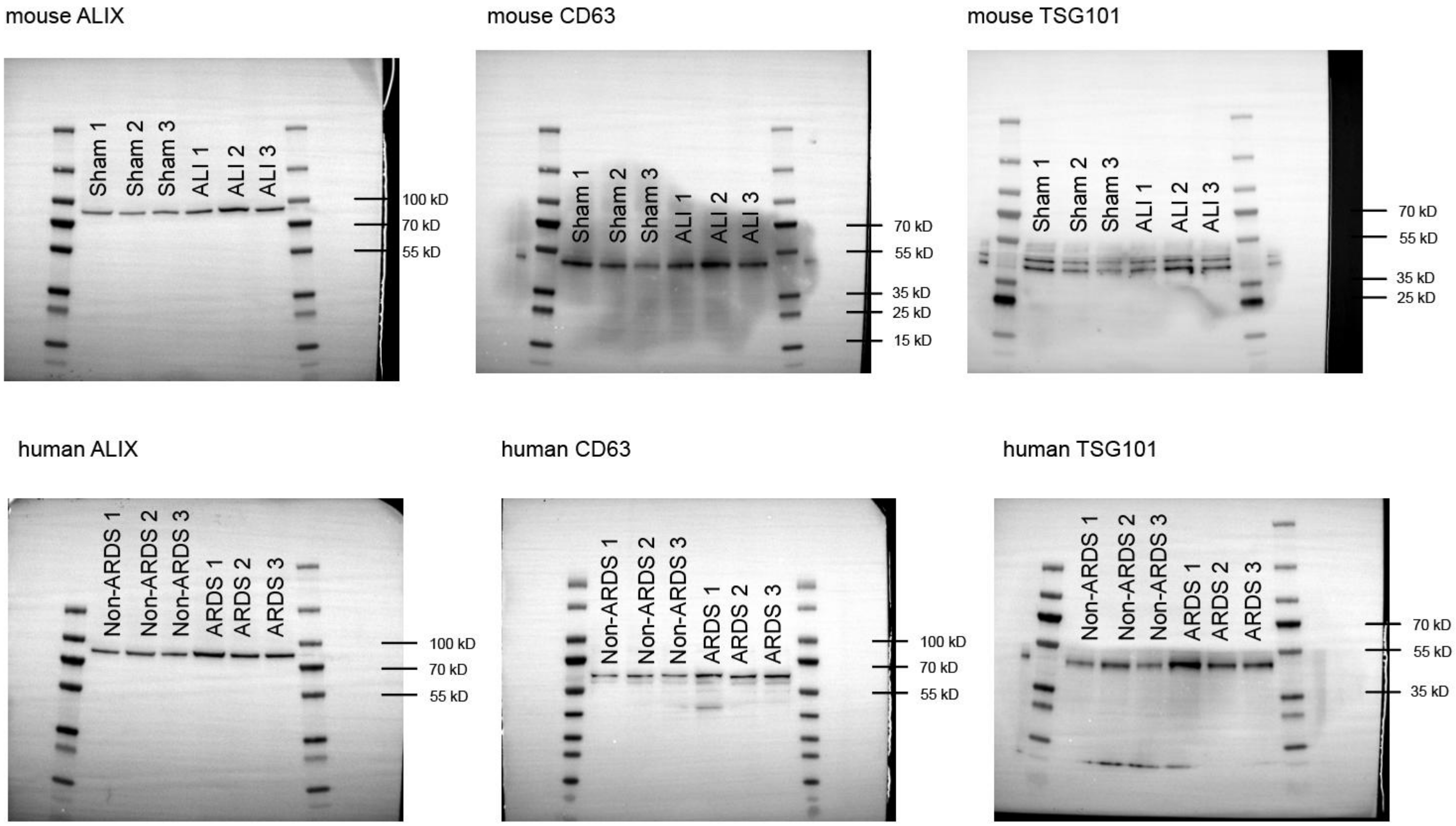
mouse ALIX
Sham 1
Sham 2
Sham 3
ALI 1
ALI 2
ALI 3
100 kD
70 kD
55 kD
mouse CD63
Sham 1
Sham 2
Sham 3
ALI 1
ALI 2
ALI 3
70 kD
55 kD
35 kD
25 kD
15 kD
mouse TSG101
Sham 1
Sham 2
Sham 3
ALI 1
ALI 2
ALI 3
70 kD
55 kD
35 kD
25 kD
human ALIX
Non-ARDS 1
Non-ARDS 2
Non-ARDS 3
ARDS 1
ARDS 2
ARDS 3
100 kD
70 kD
55 kD
human CD63
Non-ARDS 1
Non-ARDS 2
Non-ARDS 3
ARDS 1
ARDS 2
ARDS 3
100 kD
70 kD
55 kD
human TSG101
Non-ARDS 1
Non-ARDS 2
Non-ARDS 3
ARDS 1
ARDS 2
ARDS 3
70 kD
55 kD
35 kD

Figure 2C

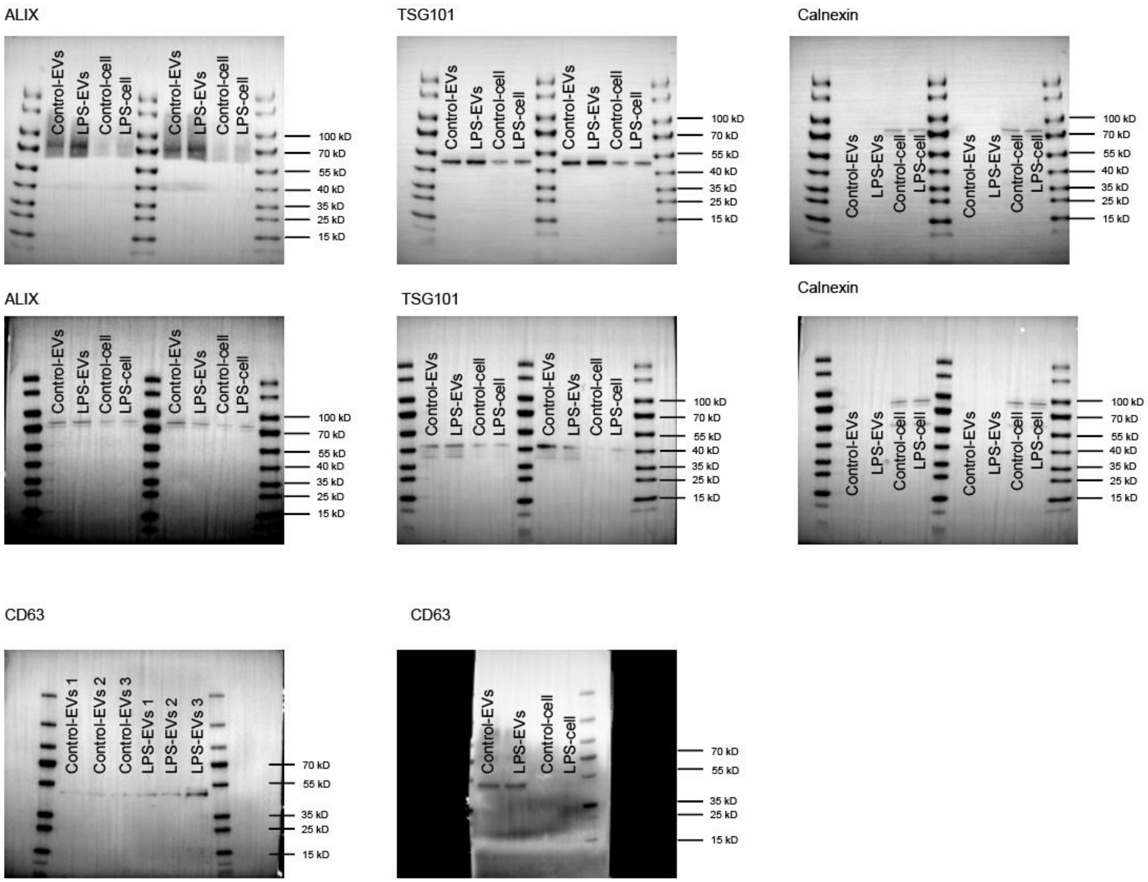
ALIX
Control-EVs
LPS-EVs
Control-cell
LPS-cell
100 kD
70 kD
55 kD
40 kD
35 kD
25 kD
15 kD
TSG101
Calnexin
CD63
Control-EVs 1
Control-EVs 2
Control-EVs 3
LPS-EVs 1
LPS-EVs 2
LPS-EVs 3

Figure 3K

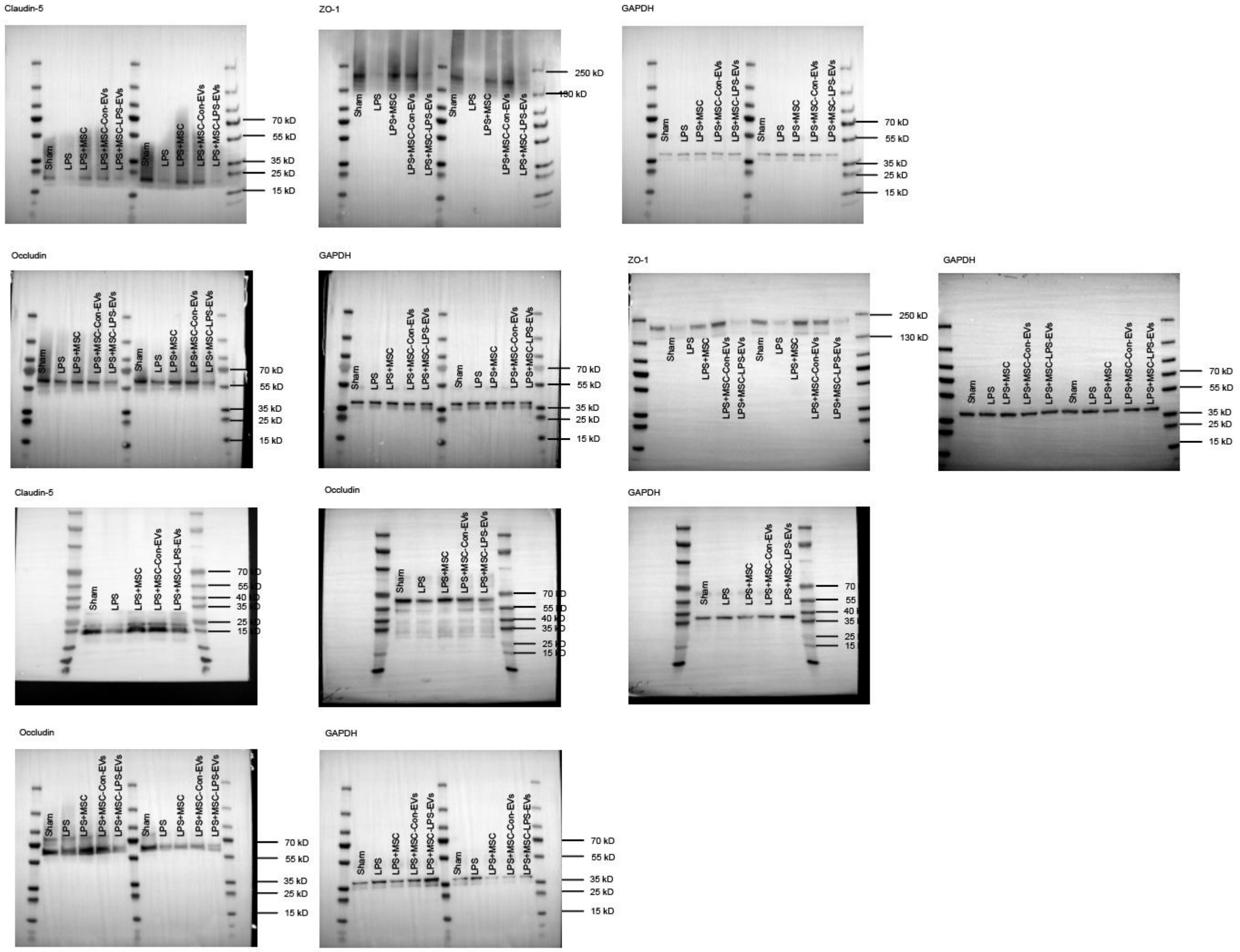
Claudin-5
ZO-1
GAPDH
Occludin
GAPDH
ZO-1
GAPDH
Claudin-5
Occludin
GAPDH
Occludin
GAPDH
Sham
LPS
LPS+MSC
LPS+MSC-Con-EVs
LPS+MSC-LPS-EVs
250 kD
130 kD
70 kD
55 kD
40 kD
35 kD
25 kD
15 kD

Figure 3O

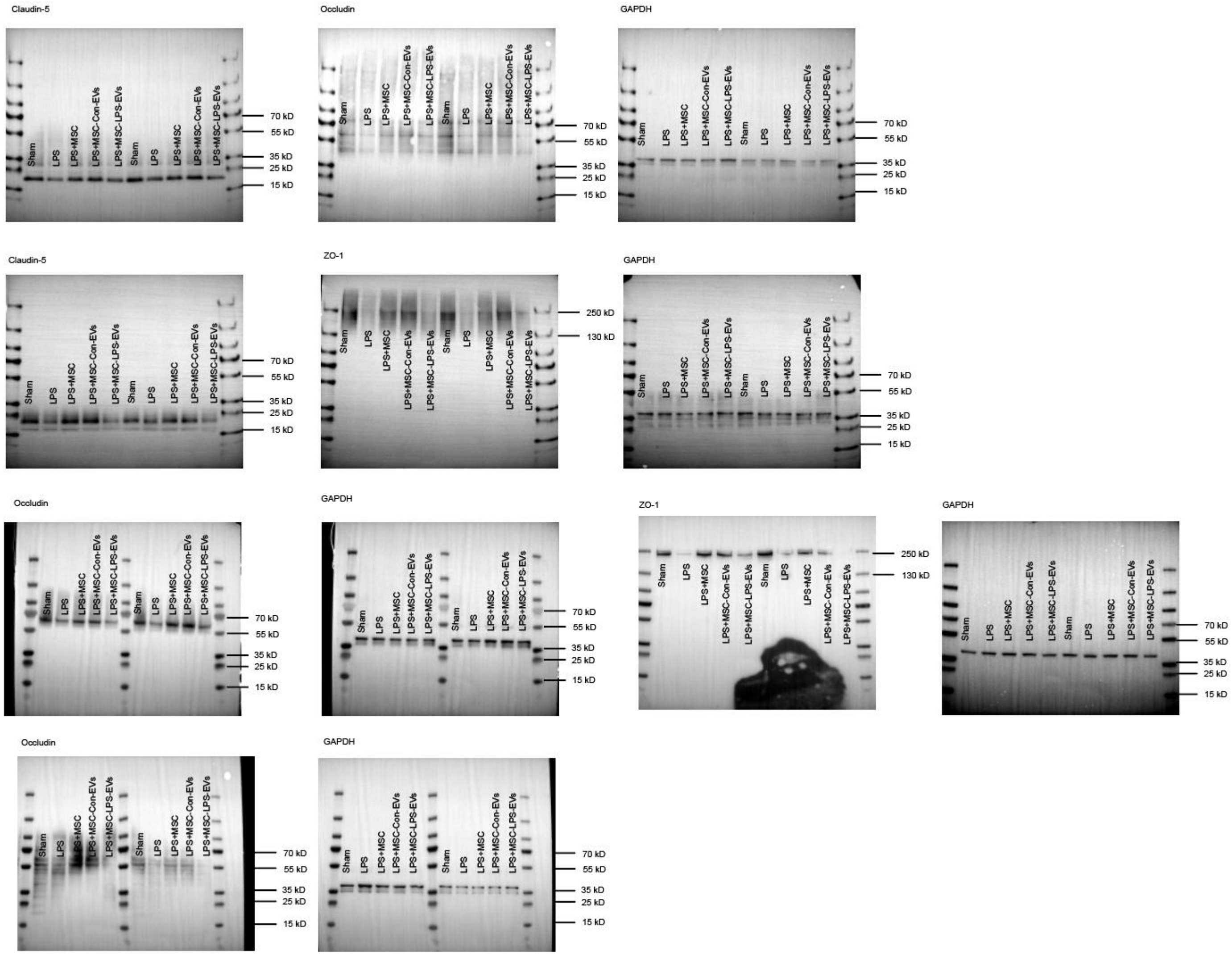

Claudin-5
Occludin
GAPDH
ZO-1
Sham
LPS
LPS+MSC
LPS+MSC-Con-EVs
LPS+MSC-LPS-EVs
250 kD
130 kD
70 kD
55 kD
35 kD
25 kD
15 kD

Figure 5D

IDH2

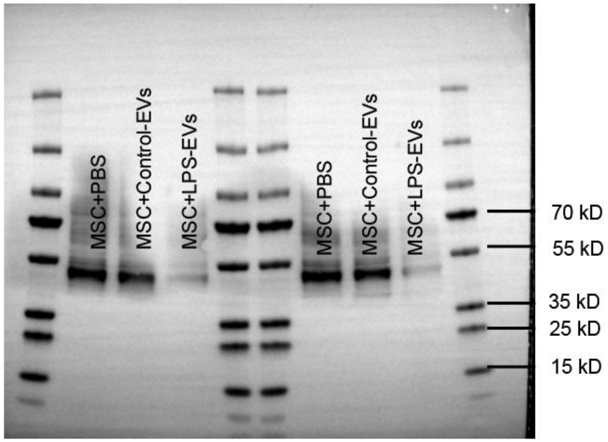

GAPDH

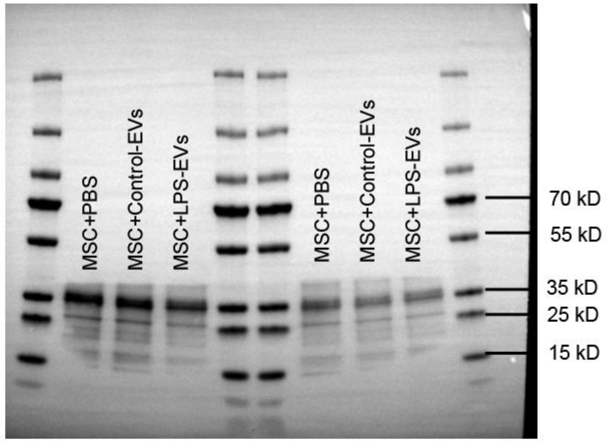

IDH2

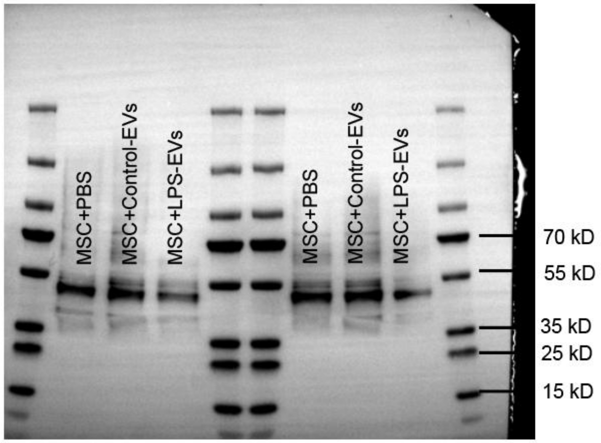

GAPDH

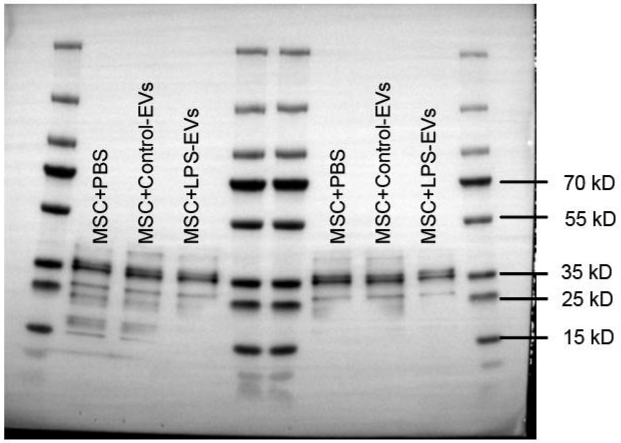

Figure 8G

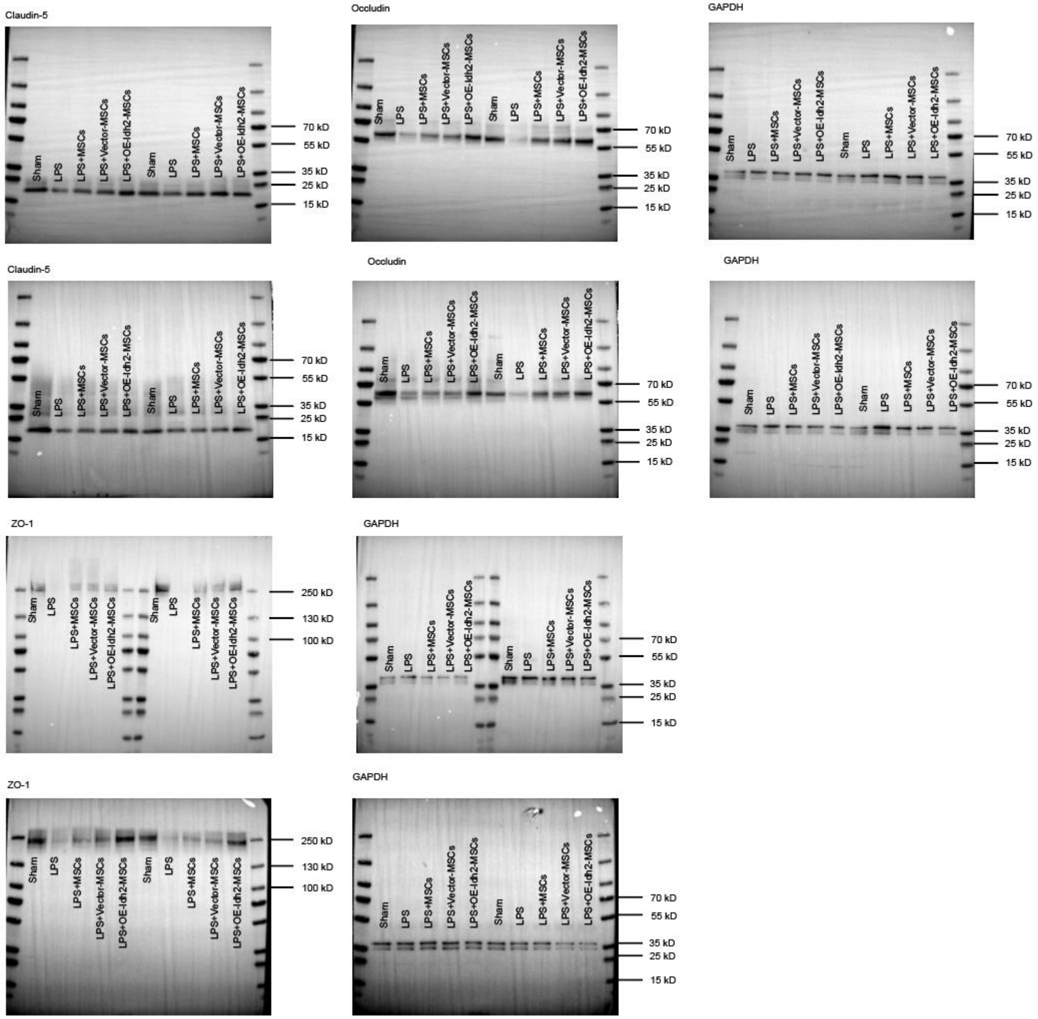

Claudin-5
Sham
LPS
LPS+MSCs
LPS+Vector-MSCs
LPS+OE-Idh2-MSCs
70 kD
55 kD
35 kD
25 kD
15 kD
Occludin
GAPDH
ZO-1
250 kD
130 kD
100 kD
