# Supplementary material for: Endothelial cell-derived extracellular vesicles modulate the therapeutic efficacy of mesenchymal stem cells through IDH2/TET pathway in ARDS
Source: Cell Commun Signal. 2024 May 27;22:293. doi: 10.1186/s12964-024-01672-0 (PMC11129421; doi:10.1186/s12964-024-01672-0)
Supplement: Supplementary file 3 — Supplementary Material 3 [file 12964_2024_1672_MOESM3_ESM.docx]

**Supporting Information**

**1 Supplementary Materials and Methods**

**BM-MSCs characterization**

Osteogenic, adipogenic, and chondrogenic differentiation abilities of BM-MSCs were respectively identified by mMSC osteogenic differentiation kit (MUXMX-90021, China), adipogenic differentiation kit (MUXMX-90031, China) and chondrogenic differentiation kit (MUXMX-90041, China) according to the instructions.

**Cell culture**

MLE-12 cells were maintained in DMEM-F12 (Gibco, USA) supplemented with 10% FBS (ExCell, China), 1% penicillin and streptomycin (Gibco, USA) and incubated at 37 °C in 5% CO2.

**Detection of LPS concentration**

LPS concentrations in LPS-EVs samples with different particle numbers were detected using a bacterial LPS concentration detection kit (MEIMIAN, China). A second round of ultracentrifugation (200,000 × g at 4 °C for 2 hours) was performed to precipitate EVs, and the supernatant of the LPS-EVs group after the second ultracentrifugation was taken as the positive control.

**2** **Supplementary Figures**


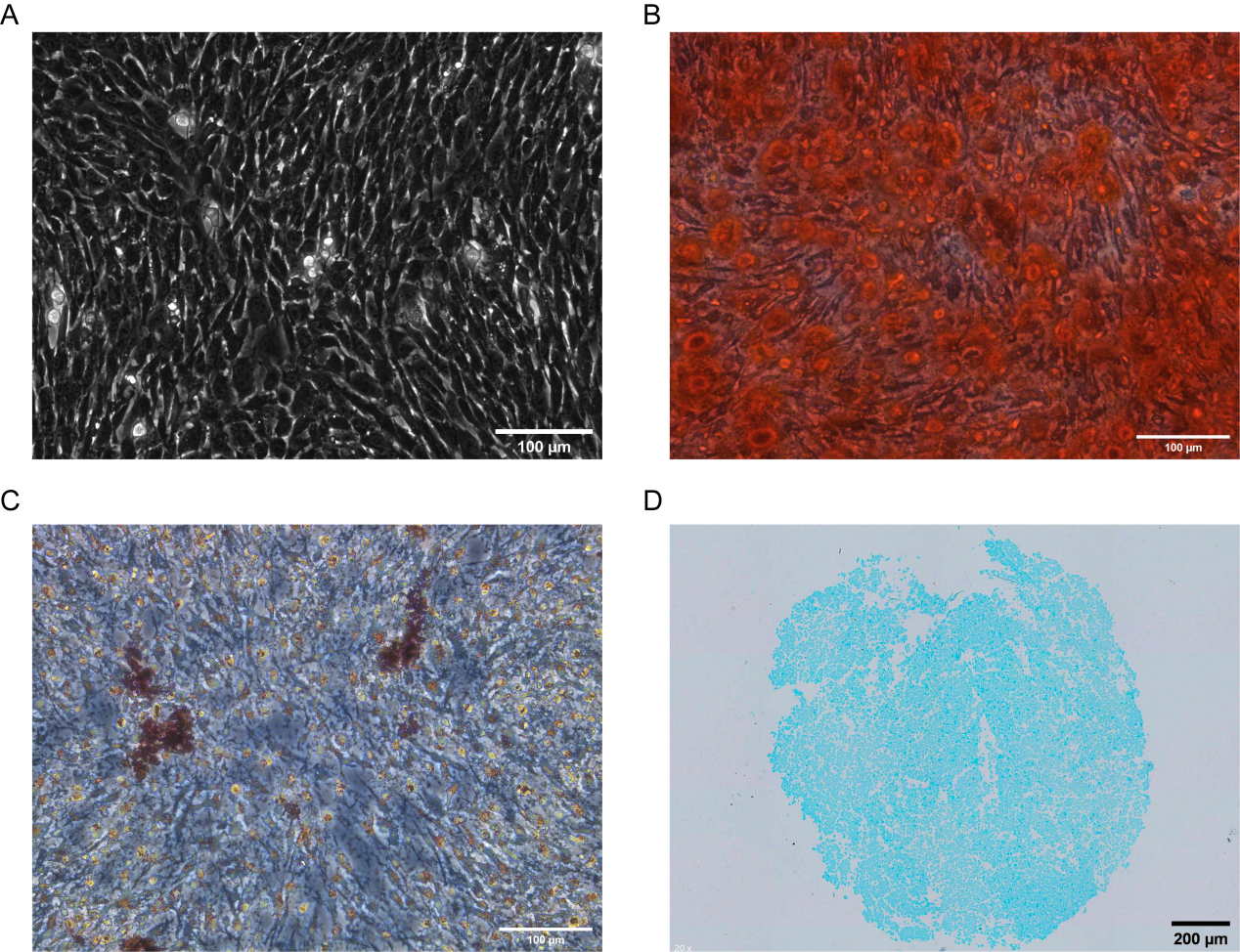


**Fig. S1** Observation of the morphology and identification of differentiation capabilities of BM-MSCs. **A** Morphological observation of BM-MSCs. Scale bar: 100 μm. **B** Osteogenic differentiation of BM-MSCs. Scale bar: 100 μm. **C** Adipogenic differentiation of BM-MSCs. Scale bar: 100 μm. **D** Chondrogenic differentiation of BM-MSCs. Scale bar: 200 μm.


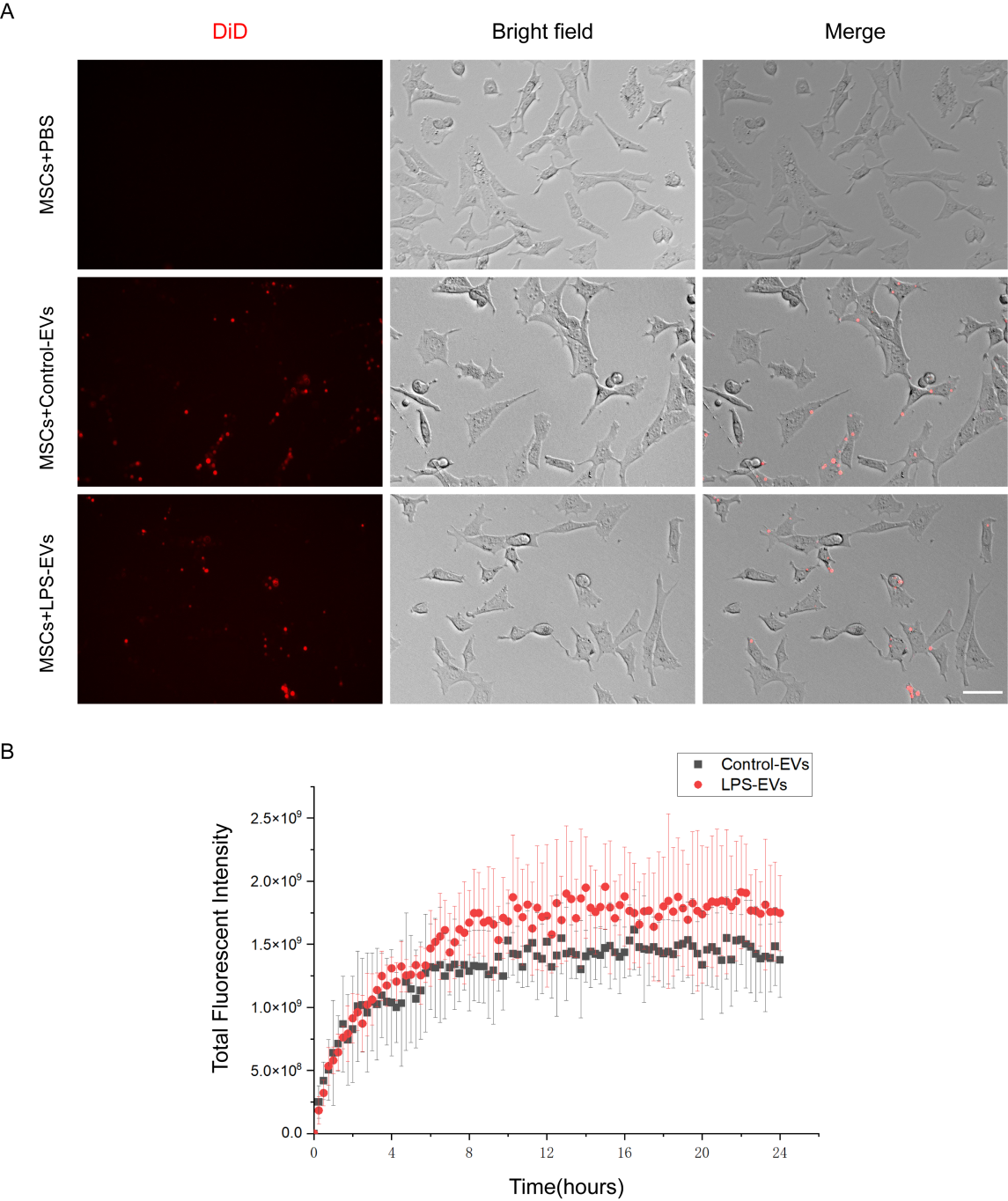


**Fig. S2** Dynamic uptake assay shows the ability of of BM-MSCs to take up Control-EVs and LPS-EVs. **A** Representative images of DiD-labeled iMPMEC-EVs uptake by BM-MSCs. Scale bar: 50 μm. **B** Cumulative fluorescent intensity of DiD-labeled iMPMEC-EVs uptake by BM-MSCs over a 24-hour period. (*n* = 3).


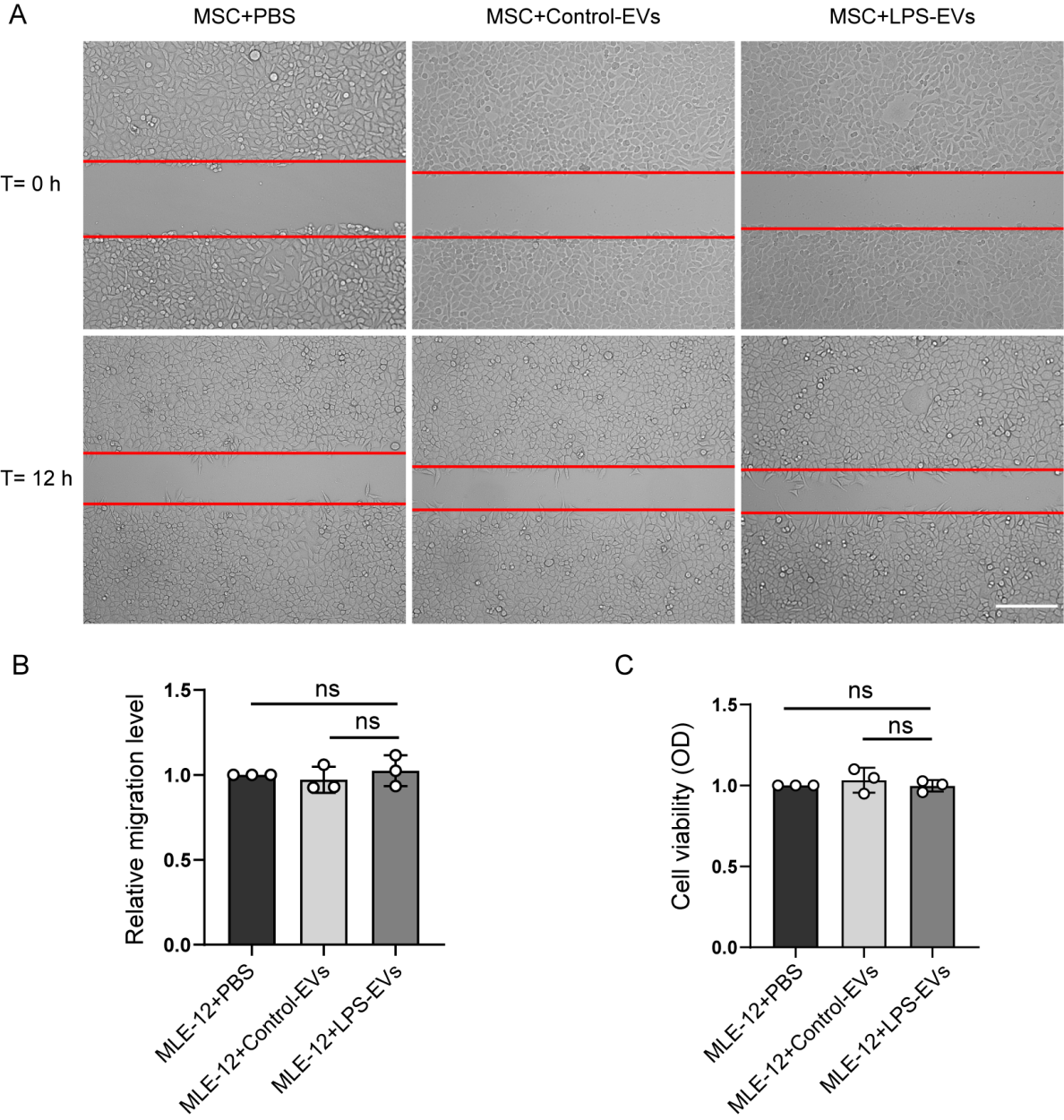


**Fig. S3** The effect of EV treatment on the viability and migration of MLE-12. The migration of MLE-12 was examined by in vitro scratch assay. **A-B** The wound areas were photographed at 0 and 12 hours and quantified by measuring the wound area in each group. (*n* = 3). Scale bar: 200 μm. **C** Cell viability of MLE-12 incubated with Control-EVs or LPS-EVs was measured using the CCK-8 assay. (*n* = 3). Data are presented as mean ± SD using one-way ANOVA followed by the Tukey’s multiple comparisons test.

**
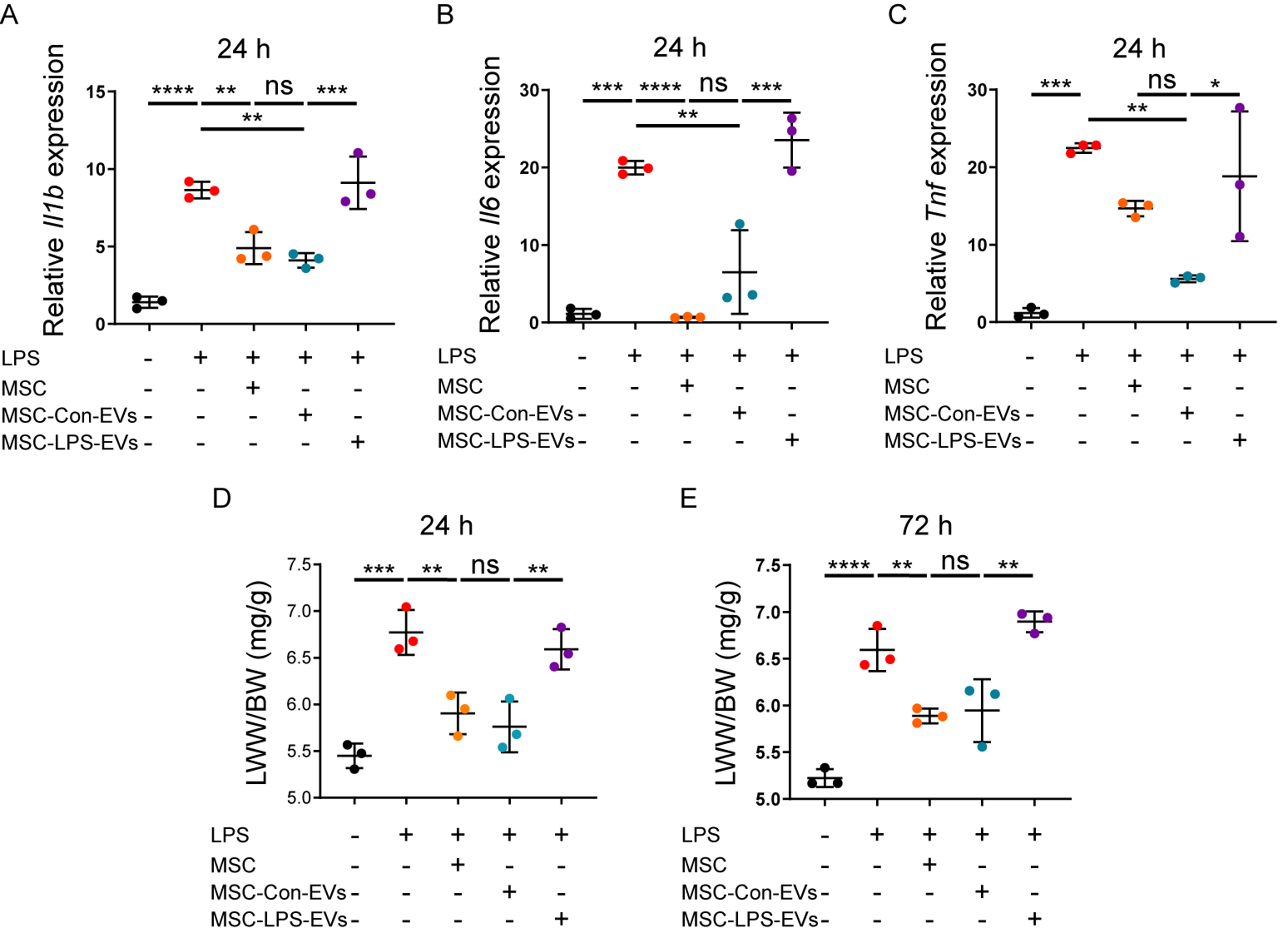
**

**Fig. S4** LPS-EVs weaken the repair effect of BM-MSCs in ALI mice. **A-C** *Il1b*, *Il6*, and *Tnf* expression 24 hours post BM-MSCs treatment in the lung tissue of ALI mice were measured using RT-qPCR. (*n* = 3). **D** The ratios of LWW/BW 24 hours post BM-MSCs treatment in the lung tissue of ALI mice. (*n* = 3). **E** The ratios of LWW/BW 72 hours post BM-MSCs treatment in the lung tissue of ALI mice. (*n* = 3). Data are presented as mean ± SD using one-way ANOVA followed by the Tukey’s multiple comparisons test. **P*＜0.05, ***P*＜0.01, ****P*＜0.001, *****P*<0.0001.

**
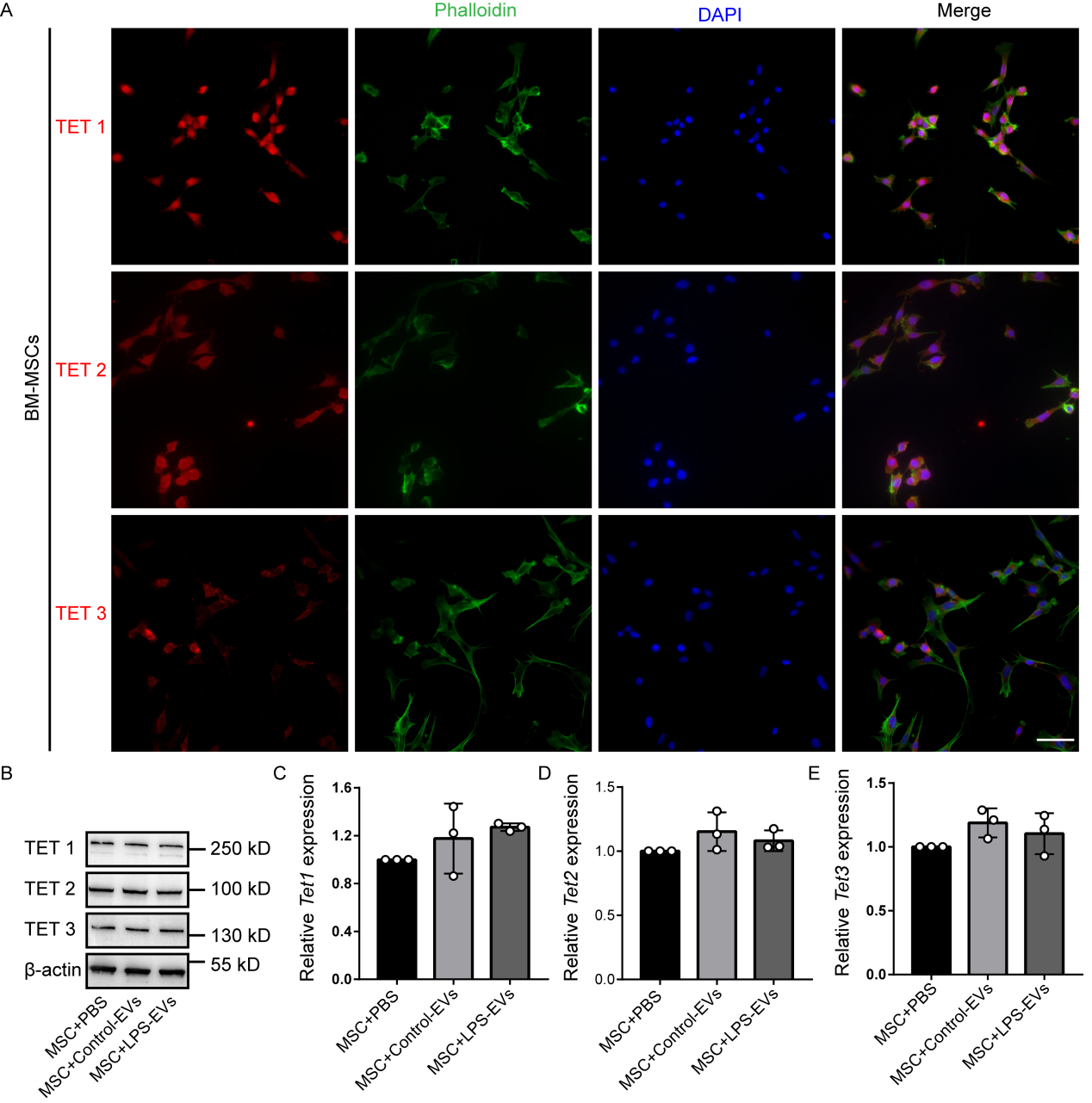
**

**Fig. S5** LPS-EVs have no effect on *Tet1*, *Tet2*, or *Tet3* expression in BM-MSCs. **A** Immunofluorescence staining demonstrates the expression of TET1, TET2, and TET3 in BM-MSCs. (*n* = 3). Scale bar: 50 μm. **B** Representative western blot of TET1, TET2, and TET3 in BM-MSCs incubated with Control-EVs or LPS-EVs for 24 hours. (*n* = 3). **C-E** *Tet1*, *Tet2*, and *Tet3* expressions in BM-MSCs after treated with Control-EVs or LPS-EVs for 24 hours were measured using RT-qPCR. (*n* = 3). Data are presented as mean ± SD using one-way ANOVA followed by the Tukey’s multiple comparisons test.


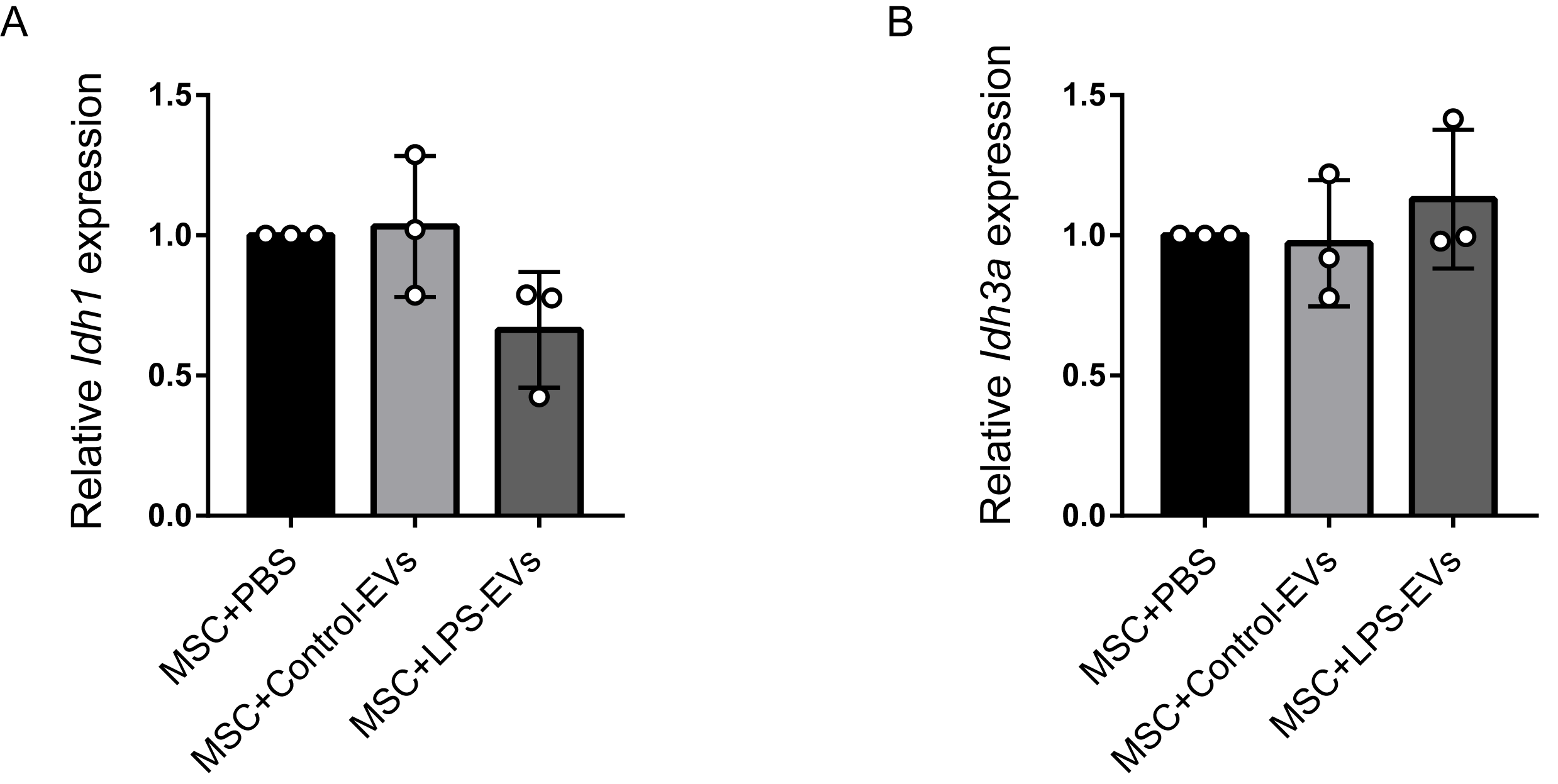


**Fig. S6** LPS-EVs have no effect on *Idh1* and *Idh3a* expression of BM-MSCs. *Idh1* **(A)** and *Idh3a* **(B)** expressions in BM-MSCs after treated with Control-EVs and LPS-EVs for 24 hours were measured using RT-qPCR. (*n* = 3). Data are presented as mean ± SD using one-way ANOVA followed by the Tukey’s multiple comparisons test.


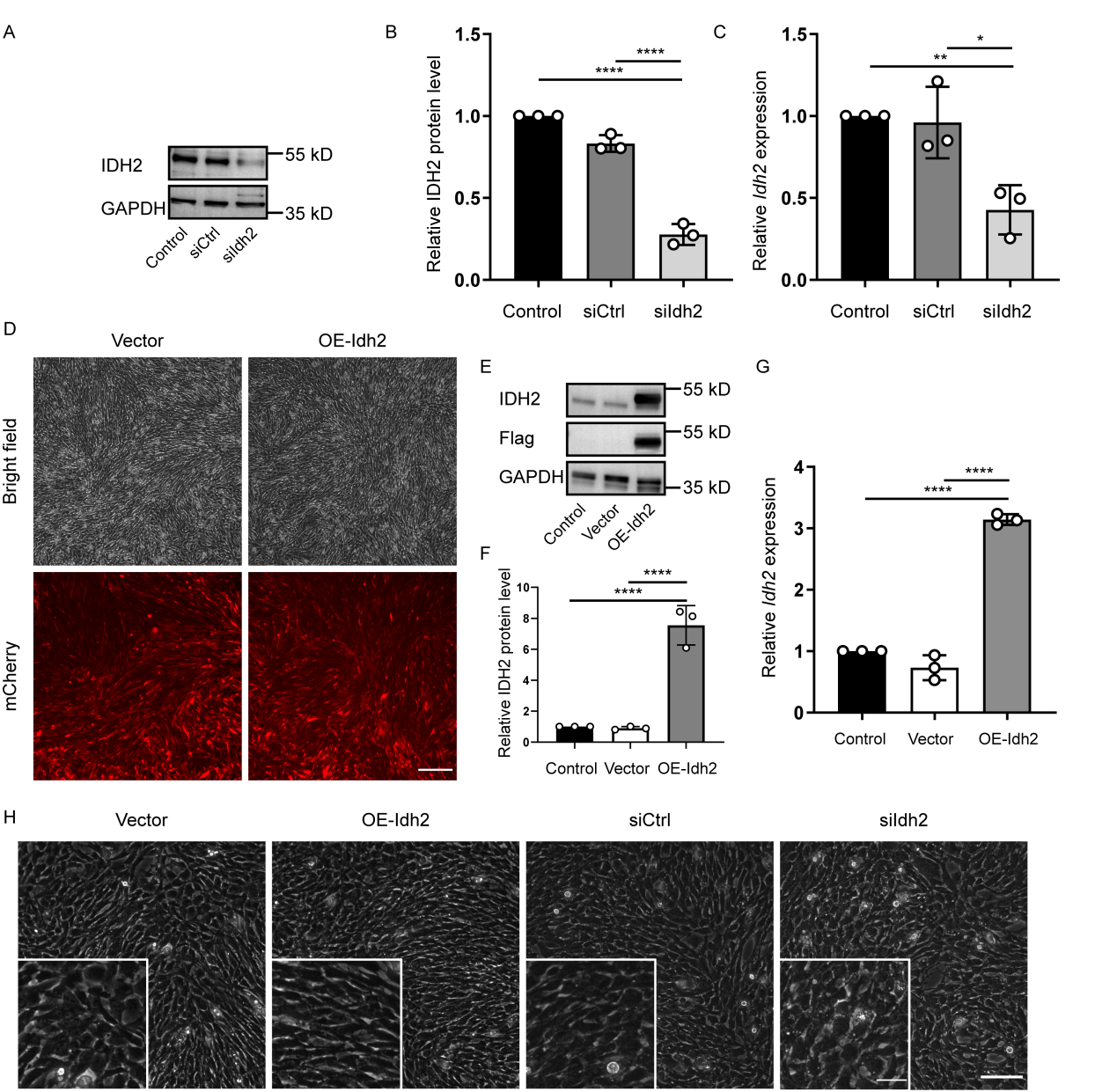


**Fig. S7** Measurement of *Idh2* expression in BM-MSCs after *Idh2* gene knockdown or overexpression. Representative western blot **(A)** and quantitative analysis **(B)** of IDH2 protein levels in the Control, siCtrl, and siIdh2 groups. (*n* = 3). **C** *Idh2* expression in the Control, siCtrl, and siIdh2 groups in BM-MSCs. (*n* = 3). **D** Representative fluorescence images of Vector-MSCs and OE-Idh2-MSCs. Scale bar: 50 μm. Representative western blot **(E)** and quantitative analysis **(F)** of IDH2 protein levels in the Control, Vector, and OE-Idh2 groups in BM-MSCs. (*n* = 3). **G** *Idh2* expression in the Control, Vector, and OE-Idh2 groups. (*n* = 3). **H** Representative images of BM-MSCs after *Idh2* gene knockdown or overexpression. (*n* = 3). Scale bar: 100 μm and 25 μm (insert). Data are presented as mean ± SD using one-way ANOVA followed by the Tukey’s multiple comparisons test. **P*＜0.05, ***P*＜0.01, *****P*＜0.0001.

**
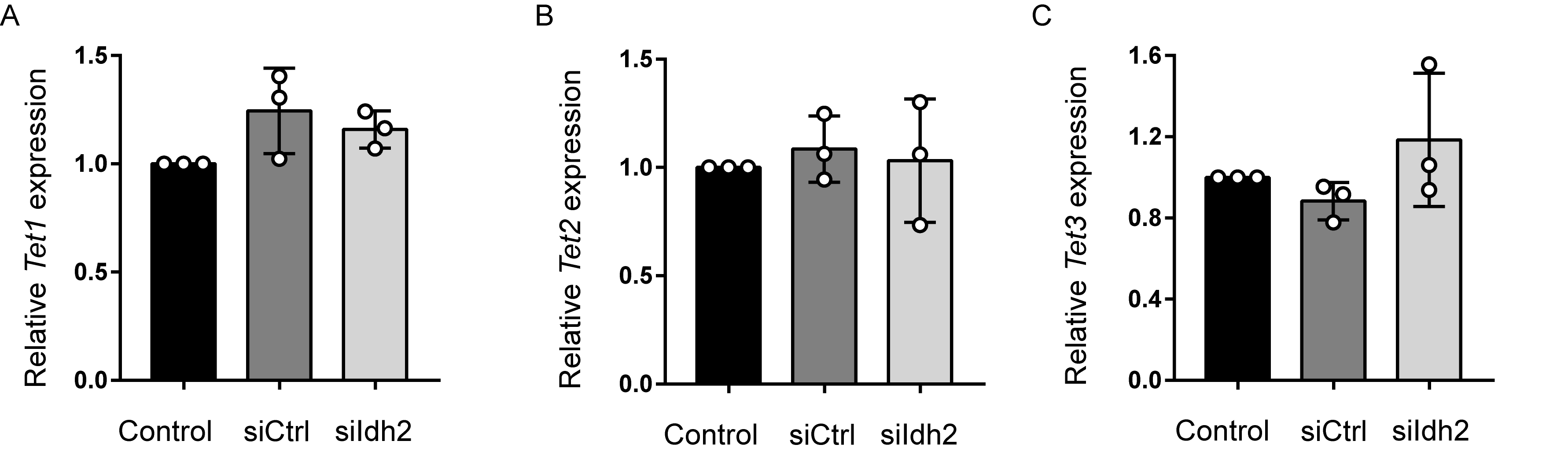
**

**Fig. S8** Knockdown of *Idh2* in BM-MSCs has no effect on *Tet1*, *Tet2*, or *Tet3* expression. *Tet1* **(A)**, *Tet2* **(B)**, and *Tet3* **(C)** expressions in BM-MSCs after treatment with siCtrl and siIdh2 for 48 hours were measured using RT-qPCR. (*n* = 3). Data are presented as mean ± SD using one-way ANOVA followed by the Tukey’s multiple comparisons test.


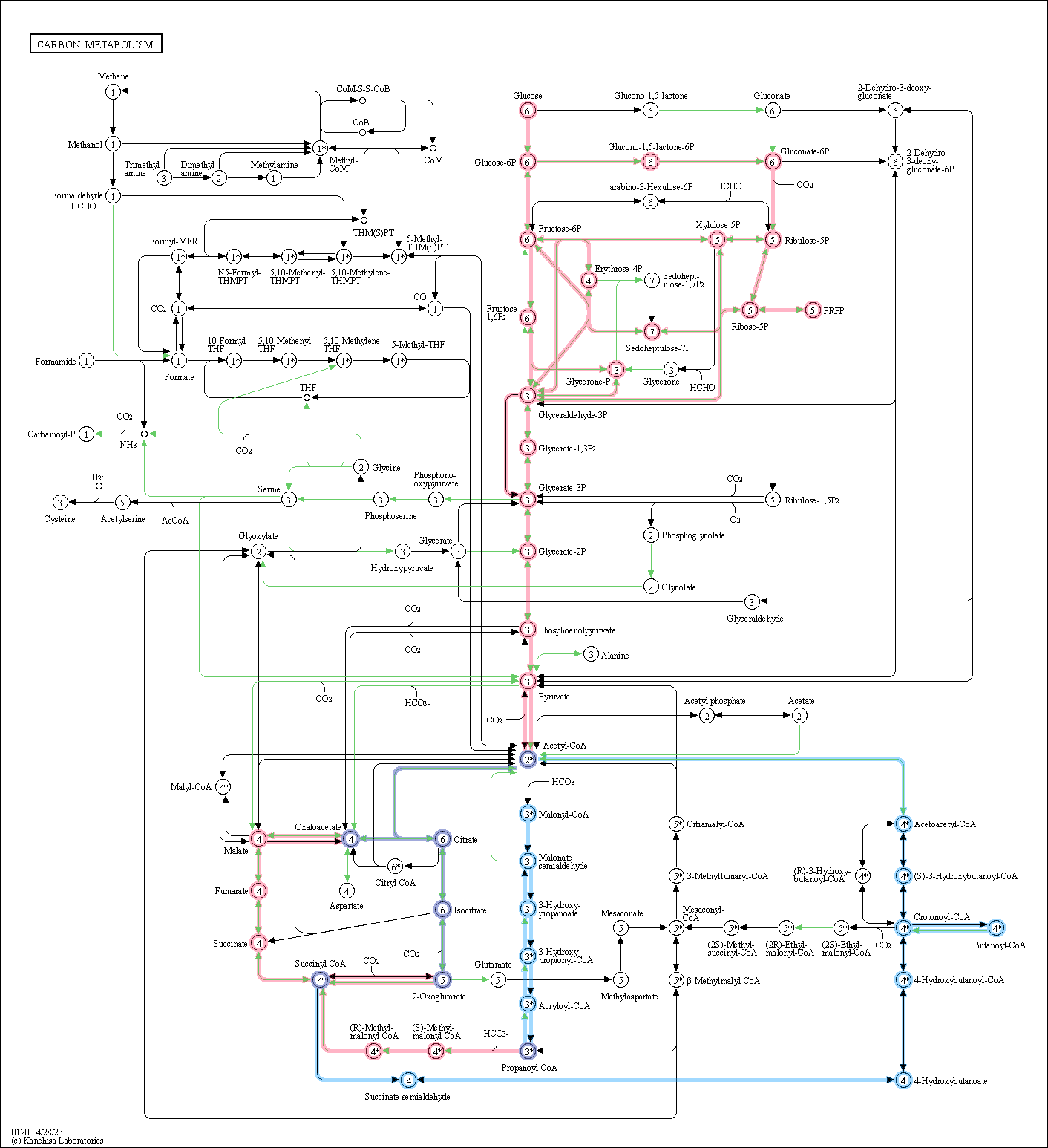


**Fig. S9 Pathways enriched by DEGs in carbon metabolism.**

**3 Supplementary Tables**

**Supplementary Table 1.** Antibodies used in this study.

| Antibody | Source | Catalog number |
| --- | --- | --- |
| TSG101 | Santa Cruz | sc-6037 |
| CD63 | Abcam | Ab217345 |
| CD63 | Abcam | Ab134045 |
| Calnexin | Cell Signaling Technology | 2679S |
| ALIX | Abcam | ab88743 |
| ZO-1 | Proteintech | 21773-1-AP |
| Occludin | Abcam | ab216327 |
| Claudin-5 | Abcam | ab131259 |
| TET1 | ABclonal | A1506 |
| TET2 | Proteintech | 21207-1-AP |
| TET3 | ABclonal | A7612 |
| 5-hmC | Abcam | Ab214728 |
| IDH2 | Proteintech | 15932-1-AP |
| GAPDH | Proteintech | 60004-Ig |
| Beta Actin | Proteintech | 66009-1-Ig |
| HRP-conjugated Affinipure Goat Anti Mouse IgG(H+L) | Proteintech | SA00001-1 |
| HRP-conjugated Affinipure Goat Anti Rabbit IgG(H+L) | Proteintech | SA00001-2 |
| Alexa Fluor 555-conjugated goat anti-rabbit IgG (H+L) | Invitrogen | A32732 |
| FITC anti-mouse CD31 | Biolegend | 102405 |
| FITC anti-human CD31 | Biolegend | 303103 |

**Supplementary Table 2.** Primer sequences used in this study.

| Gene | Forward primer | Reverse primer | usage |
| --- | --- | --- | --- |
| *Il1b* | GAAATGCCACCTTTTGACAGTG | TGGATGCTCTCATCAGGACAG | qPCR |
| *Il6* | CTGCAAGAGACTTCCATCCAG | AGTGGTATAGACAGGTCTGTTGG | qPCR |
| *Tnf* | CAGGCGGTGCCTATGTCTC | CGATCACCCCGAAGTTCAGTAG | qPCR |
| *Tet1* | TGGGAGAGCTCCCTTTGATGGTTT | TTGGGTCAATTGTGCTGCGACATC | qPCR |
| *Tet2* | TGTTGTTGTCAGGGTGAGAATC | TCTTGCTTCTGGCAAACTTACA | qPCR |
| *Tet3* | AACCAGAACGCCAAGGTCAGTAGT | TTGATCTTCTCTGGCGTGCTCAGT | qPCR |
| *Idh1* | GAGCTCTCTTGGACCGACTTC | CAACCCTCTTCTCATCGGGG | qPCR |
| *Idh2* | GTACAACACCGACGAGTCCAT | AGCCTCAGCCTCAATTGTCT | qPCR |
| *Idh3a* | CCTTTATGCCAATGTCCGGC | ACAACCCCATCAACGATCACA | qPCR |
| *Actb* | GGCTGTATTCCCCTCCATCG | CCAGTTGGTAACAATGCCATGT | qPCR |
| *Gapdh* | CCAGTTGGTAACAATGCCATGT | CCAGTTGGTAACAATGCCATGT | qPCR |

**Supplementary Table 3.** Sequences of Small interfering RNA (siRNA).

| Gene | Name | Sequence |
| --- | --- | --- |
| Control | siCtrl | sense (5’ to 3’)  UUCUCCGAACGUGUCACGUTT |
|  |  | antisense (5’ to 3’)  ACGUGACACGUUCGGAGAATT |
| *Idh2* | siIdh2 | sense (5’ to 3’)  GGUAUGAACAUCGGCUCAUTT |
|  |  | antisense (5’ to 3’)  AUGAGCCGAUGUUCAUACCTT |

**Supplementary Table 4.** Clinical characteristics of patients with ARDS and non-ARDS controls

| **characteristic** | **Non-ARDS (*n* = 4)** | **ARDS (*n* = 5)** |
| --- | --- | --- |
| Age (year) | 60.5 ± 23.5 | 72.0 ± 5.7 |
| Gender, male, n (%) | 0 (0.0) | 4 (80.0) |
| Cause of pneumonia |  |  |
| Gram-negative bacilli | - | 2 (40.0) |
| Gram-positive bacilli | - | 1 (20.0) |
| Unknow | - | 2 (40.0) |
| SOFA score | 3.5 ± 1.0 | 7.4 ± 2.1 |
| PaO_2_/FiO_2_ (mmHg) | 360.4 ± 43.4 | 199.9 ± 48.9 |
| ICU length of stay (day) | 16.3 ± 18.9 | 21.4 ± 11.3 |
| Mortality, n (%) | 0 (0.0) | 1 (20.0) |

Categorical variables are expressed as n (%), and continuous variables are presented as the mean ± SD. ICU, intensive care unit; PaO_2_/FiO_2_, the ratio of partial pressure of oxygen of fractional inspired oxygen; SOFA, sequential organ failure assessment.

**Supplementary Table 5. The concentrations of LPS in the LPS-EV samples**

| Group | EVs (particles/ml) | LPS concentration (EU/ml) |
| --- | --- | --- |
| Control-EVs | 10^8 | <0.001 |
|  | 10^8 | <0.001 |
|  | 10^8 | <0.001 |
| LPS-EVs | 10^8 | <0.001 |
|  | 10^8 | <0.001 |
|  | 10^8 | <0.001 |
|  | 10^9 | <0.001 |
|  | 10^9 | <0.001 |
|  | 10^9 | <0.001 |
|  | 10^10 | <0.001 |
|  | 10^10 | <0.001 |
|  | 10^10 | <0.001 |
| Positive Control | - | 0.057 |
|  | - | 0.064 |
|  | - | 0.046 |

The supernatant of the LPS-EVs group after the second ultracentrifugation was taken as the positive control.
